# The Myeloid LSECtin Is a DAP12-Coupled Receptor That Is Crucial for Inflammatory Response Induced by Ebola Virus Glycoprotein

**DOI:** 10.1371/journal.ppat.1005487

**Published:** 2016-03-04

**Authors:** Dianyuan Zhao, Xintao Han, Xuexing Zheng, Hualei Wang, Zaopeng Yang, Di Liu, Ke Han, Jing Liu, Xiaowen Wang, Wenting Yang, Qingyang Dong, Songtao Yang, Xianzhu Xia, Li Tang, Fuchu He

**Affiliations:** 1 State Key Laboratory of Proteomics, Beijing Proteome Research Center, Beijing Institute of Radiation Medicine, Beijing, China; 2 Department of Biochemistry and Molecular Biology, Anhui Medical University, Hefei, Anhui Province, China; 3 Military Veterinary Institute, Academy of Military Medical Science of PLA, Changchun, China; 4 College of Life Sciences, Peking University, Beijing, China; 5 Department of Biology Sciences and Biotechnology, Tsinghua University, Beijing, China; Mount Sinai School of Medicine, UNITED STATES

## Abstract

Fatal Ebola virus infection is characterized by a systemic inflammatory response similar to septic shock. Ebola glycoprotein (GP) is involved in this process through activating dendritic cells (DCs) and macrophages. However, the mechanism is unclear. Here, we showed that LSECtin (also known as CLEC4G) plays an important role in GP-mediated inflammatory responses in human DCs. Anti-LSECtin mAb engagement induced TNF-α and IL-6 production in DCs, whereas silencing of LSECtin abrogated this effect. Intriguingly, as a pathogen-derived ligand, Ebola GP could trigger TNF-α and IL-6 release by DCs through LSECtin. Mechanistic investigations revealed that LSECtin initiated signaling via association with a 12-kDa DNAX-activating protein (DAP12) and induced Syk activation. Mutation of key tyrosines in the DAP12 immunoreceptor tyrosine-based activation motif abrogated LSECtin-mediated signaling. Furthermore, Syk inhibitors significantly reduced the GP-triggered cytokine production in DCs. Therefore, our results demonstrate that LSECtin is required for the GP-induced inflammatory response, providing new insights into the EBOV-mediated inflammatory response.

## Author Summary

Ebola virus (EBOV), a highly virulent pathogen, causes a severe hemorrhagic fever syndrome. The fatal infection is characterized by a systemic inflammatory response similar to septic shock. Ebola glycoprotein (GP) is thought to contribute to disease pathogenesis, as high amounts of shed GP from virus-infected cells are detected in patients, and activate macrophages and dendritic cells (DCs) to produce proinflammatory cytokines. Here, we show that LSECtin plays an important role in GP-mediated inflammatory responses in human DCs. LSECtin is a DAP12-coupled receptor able to initiate specific signaling events in human DCs. LSECtin interacts with Ebola GP and results in DAP12 phosphorylation. LSECtin knockdown impairs the production of proinflammatory cytokines induced by Ebola GP. Thus, this study suggests that LSECtin may contribute to Ebola GP-mediated pathogenicity.

## Introduction

Ebola virus (EBOV), a member of the family *Filoviridae*, is the causative agent of severe hemorrhagic fever in humans, which is responsible for the outbreak in West African countries in 2014 [1]. Following EBOV infection, dendritic cells (DCs) and macrophages are the early and preferred replication sites of this virus, after which other cell types, including endothelial cells, epithelial cells and hepatocytes, are rapidly infected [2,3]. In experimental animal models, excessive production of proinflammatory cytokines and chemokines occurs during lethal EBOV infection, leading to endothelial cell permeability, multiorgan failure, and severe clotting disorders and culminating in a final septic shock-like syndrome [4–6]. More importantly, a fatal outcome in infected patients is also associated with aberrant innate immunity characterized by a “cytokine storm”, with hypersecretion of numerous proinflammatory mediators [3,7], suggesting that the inflammatory response plays an important role in EBOV pathogenesis. Consequently, identification of the molecular mechanisms of the inflammatory response is very important to our understanding of EBOV diseases.

EBOV genome consists of seven genes that encode seven structural proteins. Glycoprotein (GP) gene is the fourth of seven genes and encodes type I transmembrane GP termed pre-GP via transcriptional RNA editing [8,9]. The pre-GP is cleaved by furin into two subunits, GP1 and GP2, which remain linked by a disulfide bond [10,11]. This heterodimer (GP_1,2_) is known to form a trimer on the viral surface. The cleavage of surface GP by tumor necrosis factor-α-converting enzyme (TACE) releases a trimeric GP, termed shed GP [12]. During EBOV infection, significant amounts of shed GP can be detected [12]. Recently, it has been demonstrated that shed GP can induce the production of proinflammatory cytokines by activating non-infected DCs and macrophages, which can explain the dysregulated inflammatory host reactions to Ebola infection [13]. In addition, Ebola virus-like particles (eVLPs) consisting of virus protein (VP40) and GP are able to induce the activation of DCs [14]. GP is required for eVLPs to induce DCs cytokine production [15,16]. All of these results support that GP can induce inflammatory response. However, the molecular mechanism underlying GP-mediated inflammatory responses is unclear.

Inflammatory responses are rapidly elicited in response to infection by pathogens [17,18]. Innate immune cells including macrophages and DCs play important roles in this process. DCs and macrophages express diverse pattern recognition receptors (PRRs) that recognize conserved pathogen-associated molecular patterns (PAMPs) to elicit inflammatory immune responses via upregulation of proinflammatory cytokines such as tumor necrosis factor (TNF) and IL-6. C-type lectin receptors (CLRs) have been identified as PRRs and play important roles in initiating an innate immune response [19]. The functions of these receptors in immunity as PRRs for carbohydrates present on fungi and some bacterial have been well defined, such as via dectin-1 and DC-SIGN, which signal through Syk [20] and Raf-1 [21], respectively. However, the role of CLRs in inflammation mediated by virus components is less documented. The lectin LSECtin is encoded in the same chromosomal locus as DC-SIGN and also expressed by human peripheral blood DCs as well as DCs and macrophages generated in vitro [22,23]. It has been reported that LSECtin binds exogenous Ebola GP [24,25] and mediates its internalization as a PRR [23]. However, it is unclear whether LSECtin initiates specific signaling events and is involved in GP-mediated inflammatory responses.

In this study, we report that LSECtin is a DAP12-coupled activating receptor that recognizes Ebola GP. We show that triggering of endogenous LSECtin in DCs by either its mAb or GP activates Syk and ERK and leads to CARD9- and Syk-dependent cytokine production. Collectively, these findings suggest that LSECtin functions as a DAP12-coupled receptor and acts as a functional PRR for Ebola GP.

## Results

### LSECtin on human DCs involves the Ebola GP-induced proinflammatory cytokine production

LSECtin is a C-type lectin receptor and binds Ebola GP as a pattern recognition receptor. To verify whether LSECtin interacts GP, recombinant protein GP1-Fc was prepared and subjected to Coomassie blue staining and Western blotting (S1A and S1B Fig). In addition, we found that under nonreducing conditions, recombinant protein GP1-Fc is in monomeric form (S1C Fig). Using an enzyme-linked immunosorbent assay, our data demonstrated that GP1-Fc binds LSECtin in a dose-dependent way (S1D Fig). LSECtin has a typical carbohydrate recognition domain (CRD) and binds Ebola GP in a Ca^2+^-dependent manner [25]. Amino acid sequence alignment of the CRD of LSECtin with those of other C-type lectins indicates that 2 amino acids, Asn256 and Asn274, interact with Ca^2+^ through their carbonyl groups. Thus, we mutated the residues to aspartic acid. The mutant LSECtin (N256D or N274D) did not bind Ebola GP, which suggests that these residues are critical for recognition of EBOV GP (S1D Fig). Furthermore, we also performed a cell surface staining assay and demonstrated that Jurkat cells lentivirally transfected with LSECtin rather than mutant LSECtin bind GP (S1E Fig).

**Fig 1:**
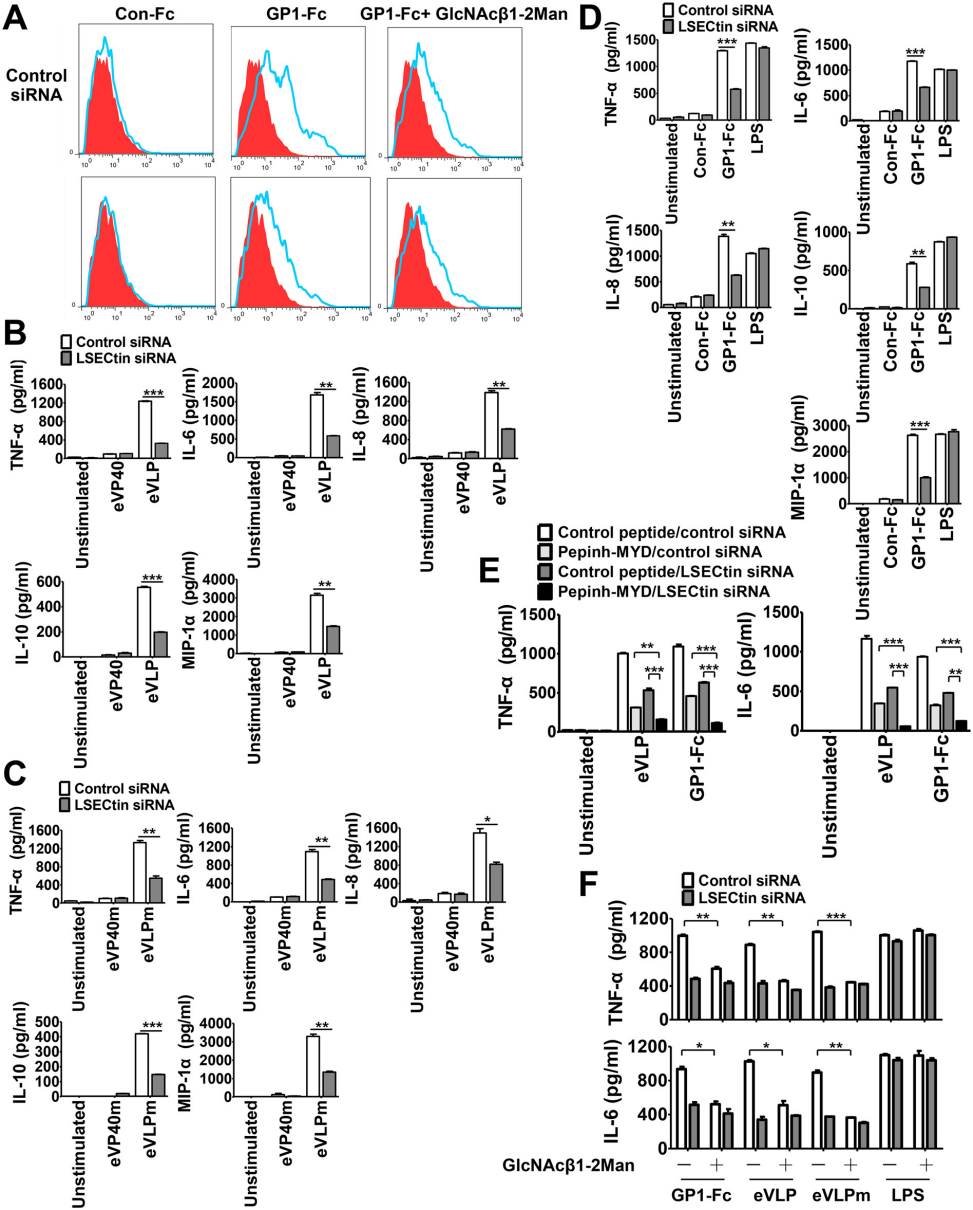
Induction of proinflammatory cytokines by eVLPs, eVLPm or GP1-Fc requires LSECtin. (A) MDDCs transfected with control siRNA or LSECtin-specific siRNA were incubated with Con-Fc, GP1-Fc or GP1-Fc plus GlcNAcβ1-2Man (200μM) in the presence of human FcR inhibitor. Bound proteins were detected by APC-conjugated mouse anti-human IgG and analyzed by flow cytometry. (B, C and D) MDDCs transfected with control siRNA or LSECtin-specific siRNA were stimulated with eVLP (B), eVLPm (C) or plate-bound GP1-Fc (D). After stimulation, the cytokine production in the supernatants was measured by ELISA. eVLP, Ebola VLPs produced in insect cells; eVLPm, Ebola VLPs produced in mammalian 293T cells. (E) MDDCs transfected with control siRNA or LSECtin-specific siRNA were pretreated with Pepinh-MYD (40μM) or control peptide (40μM) and then stimulated with eVLPs or plate-bound GP1-Fc. Cytokine production in the supernatants was measured by ELISA after overnight stimulation. (F) MDDCs transfected with control siRNA or LSECtin-specific siRNA were stimulated with eVLP, eVLPm, plate-bound GP1-Fc or LPS in the absence or presence of GlcNAcβ1-2Man (200μM). Cytokine production in the supernatants was measured by ELISA after overnight stimulation. Data are represented as mean ± SD of two independent experiments. *p < 0.05; **p < 0.01; ***p < 0.001.

Next, we explore the expression profile of LSECtin in human blood leukocytes. As shown in S2A Fig, the anti-LSECtin mAb CCB059 did not stain granulocytes, monocytes or lymphocytes. We further investigated the expression of LSECtin on monocyte-derived DCs (MDDCs) by culturing monocytes in the presence of GM-CSF and IL-4 (S2B Fig). This treatment resulted in a strong up-regulation of LSECtin. In addition, this result was confirmed by PCR and Western blotting (S2C and S2D Fig). Ebola GP interacts with LSECtin stably expressed on Jurkat cell line. It was thus of interest to investigate whether GP could also bind LSECtin on human MDDCs. To address the issue, MDDCs were transfected with siRNA specific for LSECtin or with control siRNA for 48h (S3 Fig) and stained with GP1-Fc. First, we found that Ebola GP can bind MDDCs. More importantly, the activity is partially dependent on LSECtin. These results suggest that LSECtin involves GP binding to MDDCs (Fig 1A).

To investigate whether GP/LSECtin interaction can lead to the production of proinflammatory cytokines within the human immune system, MDDCs transfected with siRNA specific for LSECtin or with control siRNA were stimulated with eVLPs and eVP40 which were produced in insect cells. Compared with the stimulation of VP40, eVLPs significantly enhanced the production of cytokines and chemokines, suggesting that GP is required for eVLPs to activate DCs. Furthermore, we found that after LSECtin “knockdown”, MDDCs stimulated with eVLPs produced less TNF-α, IL-6, IL-8, IL-10 and MIP-1α (Fig 1B). Although eVLPs produced in insect cells or mammalian 293T cells exhibit similar DC-stimulating activities [26], eVLPs were also produced in 293T mammalian cells to determine whether the data are influenced by the insect cell expression system for GP. Similar with the results shown in Fig 1B, eVLPs produced in 293T mammalian cells induced less production of TNF-α, IL-6, IL-8, IL-10 and MIP-1α in LSECtin “knockdown” MDDCs (Fig 1C). These results suggest that LSECtin involves the Ebola GP-induced cytokine production whether it was produced in insect cells or human 293T cells.

Soluble GP1-Fc did not induce cytokine production (S4 Fig), which is consistent with the previous report [15]. To simulate the configuration and multivalency of GP on eVLPs or shed GP, GP1-Fc was coated on a culture well for stimulation of MDDCs. To directly investigate the role of LSECtin in GP1-mediated proinflammatory cytokine production, control or LSECtin siRNA-transfected MDDCs were stimulated with plate-bound GP1-Fc. Similar to the results as described above, GP1-Fc-induced cytokine production was impaired in LSECtin “knockdown” DCs (Fig 1D). In addition, our results demonstrated that the production of cytokines induced by eVLPs and plate-bound GP1-Fc was inhibited by Pepinh-MYD, a MyD88 inhibitor peptide, which is consistent with the previous reports that eVLP and shed GP induced cytokine production through TLR4/MyD88 signaling (S5A and S5B Fig) [13,27]. However, the cytokine production induced by LPS is not impacted in LSECtin “knockdown” DCs, suggesting that MyD88 signaling pathway is intact (Fig 1D). Given that the production of cytokines is partially inhibited by LSECtin silencing or MyD88 inhibitory peptide, we next determined whether there is a synergistic efficacy of LSECtin silencing in combination with MyD88 inhibitory peptide. We found that MDDCs treated by double silencing produced less TNF-α and IL-6 than LSECtin silencing or MyD88 inhibitory peptide alone treated cells (Fig 1E). In addition, we also demonstrated that there is a synergistic efficacy of LSECtin and TLR4-induced cytokines after LSECtin/TLR4 double “knockdown” DCs (S6 Fig). These results suggesting that LSECtin and TLR4/MyD88 signaling collaborate to mediate inflammatory response induced Ebola GP.

GlcNAcβ1-2Man disaccharide has been demonstrated to be a specific inhibitor of interaction between LSECtin and Ebola GP [25]. Our result also demonstrated that GlcNAcβ1-2Man inhibits the GP binding to MDDCs (Fig 1A). More importantly, the production of TNF-α and IL-6 can be inhibited by the addition of GlcNAcβ1-2Man and the effect was specific for Ebola GP as LPS-induced TNF-α and IL-6 production was unaffected by the presence of the GlcNAcβ1-2Man (Fig 1F). Collectively, these results suggest that LSECtin is selectively expressed in MDDCs and involved in GP-mediated proinflammatory cytokine production.

### LSECtin crosslinking by anti-LSECtin mAb triggers proinflammatory cytokine production

**Fig 2:**
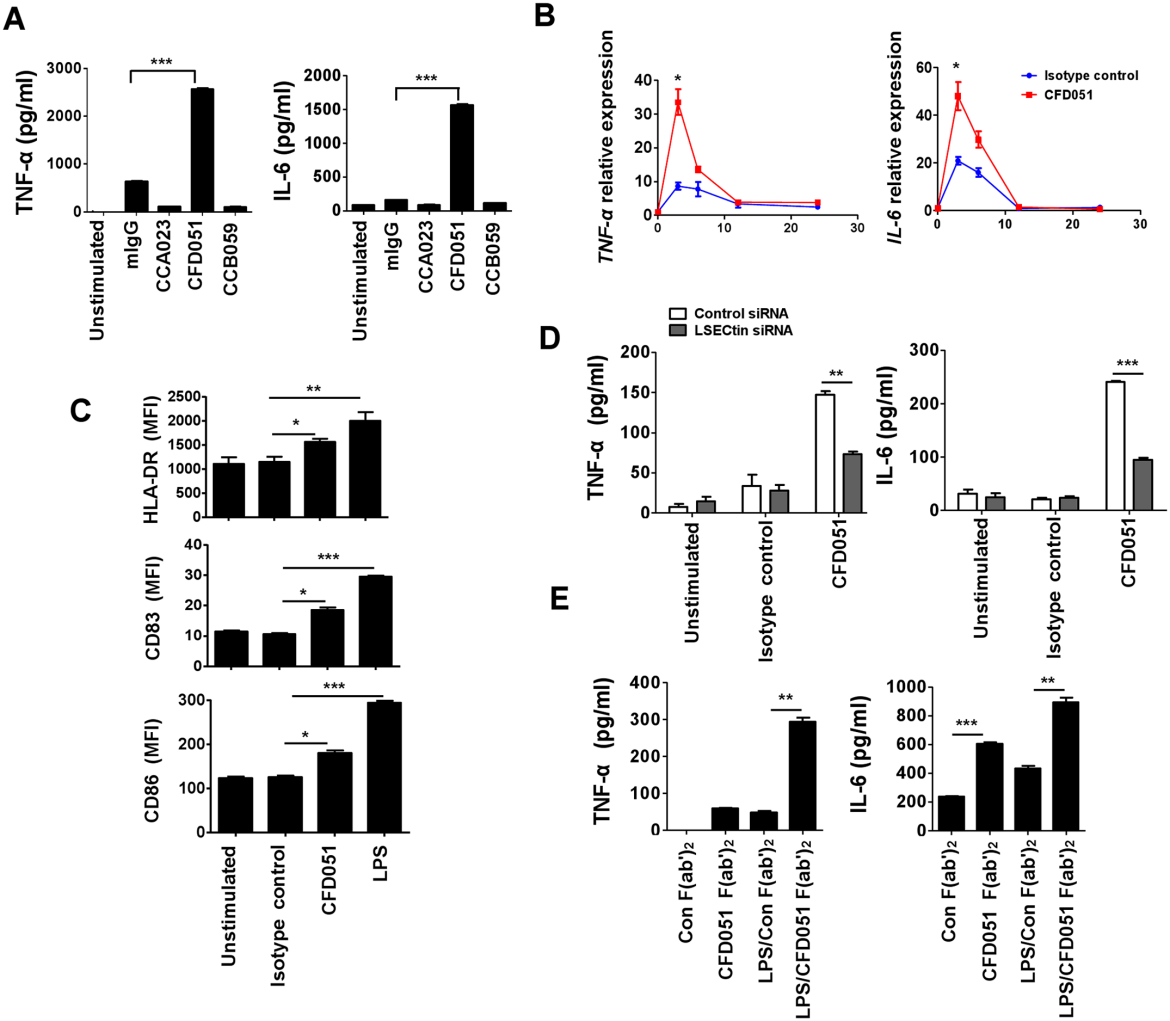
LSECtin crosslinking triggers proinflammatory cytokine production. (A) Plate-bound anti-LSECtin CFD051 mAb significantly enhanced the production of TNF-α and IL-6 by MDDCs compared with plate-bound anti-LSECtin CCA023 mAb, CCB059 mAb and mIgG isotype control. Cytokine production in the supernatants was measured by ELISA after overnight stimulation. (B) Real-time RT-PCR analysis of TNF-α and IL-6 from MDDCs seeded on control mIgG1-coated or CFD051-coated plates for the indicated times. The results are presented as the mean ± SD of triplicate wells normalized to GAPDH mRNA. (C) Flow cytometry analysis of cell surface markers on MDDCs left in medium alone or stimulated with immobilized control mIgG1 or CFD051 or with LPS (10ng/ml) for 24h. The data are presented as mean fluorescence intensity (MFI) values. (D) MDDCs were transfected with control siRNA or with LSECtin-specific siRNA for 48h and then stimulated with control mIgG1 or CFD051. After stimulation, cytokine production in the supernatants was measured by ELISA. (E) ELISA of TNF-α and IL-6 production by human MDDCs stimulated with immobilized CFD051 F(ab′)_2_ fragments in the absence or presence of LPS for 18h. Data present the mean ± SD of two independent experiments. *p < 0.05; **p < 0.01; ***p < 0.001.

The above results suggest that Ebola GP induced cytokine production by MDDCs through both LSECtin and MyD88 signaling. To specially and clearly explore the LSECtin signaling, we use anti-LSECtin mAbs, including CCA023, CFD051 and CCB059, to stimulate LSECtin signaling upon crosslinking in MDDCs. We treated MDDCs with immobilized anti-LSECtin mAbs. The production of TNF-α and IL-6 was significantly increased in MDDCs after 24h of treatment with CFD051, compared with CCA023, CCB059 and the control mIgG1, suggesting that only CFD051-LSECtin engagement promoted cytokine production (Fig 2A). We also observed similar changes in mRNA levels. We found that LSECtin engagement induced rapid but transient mRNA expression for the cytokines IL-6 and TNF-α (Fig 2B). The IL-6 and TNF-α mRNA amounts peaked approximately 3h after LSECtin ligation and subsequently declined to close to baseline 6h after LSECtin crosslinking. In addition, LSECtin engagement increased the maturation of MDDCs, as characterized by increased surface expression of HLA-DR, CD83 and CD86 (Fig 2C). Interestingly, GP/LSECtin interaction also triggers the maturation of DCs as the surface expression of CD40, CD80 and CD86 decreased in LSECtin “knockdown” DCs (S7 Fig).

To determine whether TLR signaling is involved in LSECtin-mediated cytokine production, MDDCs were pretreated with Pepinh-MYD (a MyD88 inhibitor) and stimulated the cells with plate-bound CFD051. Our results show that MyD88, a crucial adaptor of TLR signaling, was dispensable for LSECtin-mediated cytokine production (S8 Fig). To prove that the cellular effects mediated by LSECtin engagement were specific, we treated the siRNA-transfected MDDCs with CFD051 overnight. We found that after LSECtin “knockdown”, MDDCs stimulated with CFD051 produced less TNF-α and IL-6 (Fig 2D). To exclude the possibility that the TNF-α and IL-6 production was simply due to Fc receptor engagement, we prepared F(ab′)_2_ fragments of anti-LSECtin mAb and used them to stimulate MDDCs. As shown in Fig 2E, plate-bound F(ab′)_2_ fragments from anti-LSECtin mAb induced the production of TNF-α and IL-6 and markedly increased their production in the presence of LPS (a TLR4 agonist), suggesting cooperation between TLR4 and LSECtin signaling in the MDDCs.

The NF-κB factors are held in the cytoplasm in an inactive state complexed with the inhibitory IκBα proteins. Upon stimulation by LPS, IκBα is phosphorylated and subsequently degraded resulting in NF-κB activation. S9 Fig shows that LPS induced a significant reduction in IκBα that last up to 1h. The basal IκBα levels were restored by 2h. However, LPS and LSECtin mAb combined treatment induces IκBα degradation that last up to 2h. The results indicated that LSECtin and TLR4 signaling crosstalks at the level of NF-κB activation. Taken together, our results demonstrated that LSECtin engagement can specially promote TNF-α and IL-6 production and enhance the maturation of MDDCs.

### LSECtin associates with DAP12

**Fig 3:**
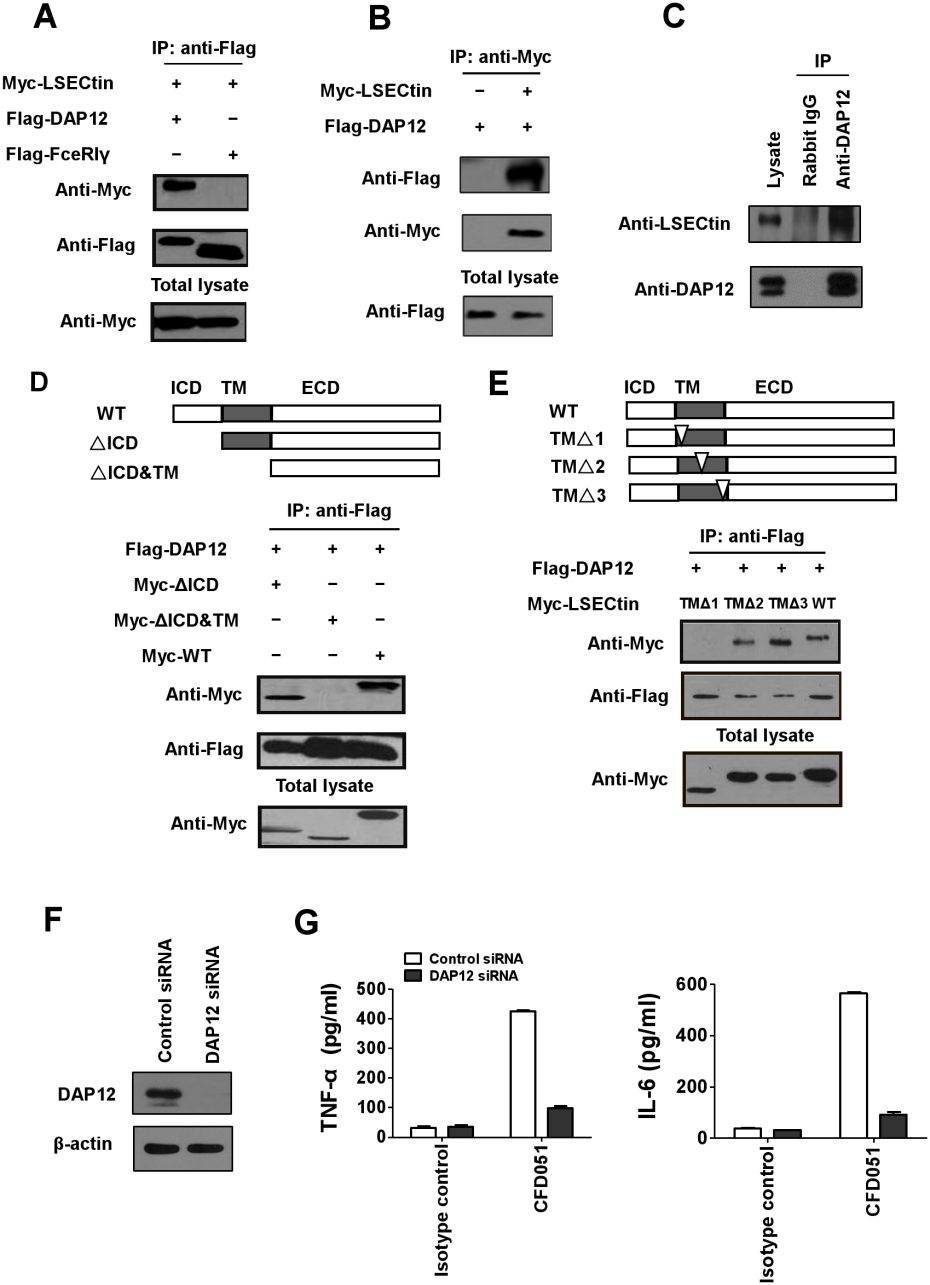
LSECtin interacts with DAP12. (A) HEK293 cells were transfected with Myc-tagged LSECtin together with Flag-tagged DAP12 or FceRIγ. Lysates IP with anti-Flag were analyzed by immunoblotting with anti-Flag and anti-Myc. (B) HEK293 cells were transfected with DAP12 alone or DAP12 combined with LSECtin. Lysates IP with anti-Myc were analyzed by immunoblotting with anti-Flag and anti-Myc. (C) Cell lysates from MDDCs were immunoprecipitated with Rabbit IgG or anti-DAP12 antibody and immunoblotted with anti-LSECtin or anti-DAP12 antibody. (D) HEK293 cells were transfected with Flag-tagged DAP12 together with different Myc-tagged LSECtin mutants, and lysates were analyzed by immunoblotting after IP with anti-Flag. The protein structures of WT LSECtin and the different mutants are shown in the diagram. WT LSECtin consists of an intracellular domain (ICD), a transmembrane domain (TM), and an extracellular domain (ECD). (E) Immunoblot of HEK293 cells transfected with Flag-tagged DAP12 together with Myc-tagged WT LSECtin or the mutant TMΔ1, TMΔ2 or TMΔ3 after IP with anti-Flag. The immunoblot was performed using anti-Flag and anti-Myc. The inverted triangle represents the location of the deletion mutants. (F) Immunoblot analysis of DAP12 expression in MDDCs 72h after transfection with control siRNA or DAP12-specific siRNA. (G) MDDCs transfected with control siRNA or DAP12-specific siRNA were stimulated with control mIgG1 or CFD051. After stimulation, cytokine production in the supernatants was measured by ELISA. Error bars represent the SD from the mean values of two independent experiments.

The above results showed that LSECtin mediates positive signaling in MDDCs. However, there is no signal transduction motif in this protein’s cytoplasmic tail. Therefore, it is likely that LSECtin is associated with an adaptor molecule to transduce signals. Co-immunoprecipitation and immunoblot analysis showed that LSECtin selectively associated with DAP12 but not with FceRIγ (Fig 3A). We also used reverse IP to show that LSECtin co-precipitated DAP12 (Fig 3B). Importantly, the interaction between endogenous LSECtin and DAP12 was also obvious in MDDCs (Fig 3C). Thus, these results suggested that LSECtin is associated with DAP12.

LSECtin does not possess any positively charged residues in the transmembrane domain that is required for the interaction with DAP12 in many other receptors. The negatively charged amino acid D50 in the transmembrane region of DAP12 is dispensable for the interaction (S10 Fig). However, the interaction of LSECtin and DAP12 was mediated through the transmembrane region of LSECtin (Fig 3D), as deficiency of the transmembrane region abolished its association with DAP12. Next, we further showed that a short stretch of transmembrane region proximal to the intracellular domain of LSECtin (amino acids 32–43) was required for association with DAP12 (Fig 3E). In addition, the interaction of LSECtin and DAP12 was independent of the only two hydrophilic threonines (T41 and T42) within the transmembrane region of LSECtin (S10 Fig).

The previous results in Fig 2 show that immobilized antibody to LSECtin can induce the production of TNF-α and IL-6. To determine whether LSECtin-mediated signaling is dependent on DAP12, MDDCs were transfected with siRNA specific for DAP12 or with control siRNA for 72 h (Fig 3F). We found that LSECtin failed to induce the production of TNF-α and IL-6 in DAP12 “knockdown” MDDCs after treatment with CFD051 antibody (Fig 3G), suggesting that LSECtin transduces signaling in a DAP12-dependent manner.

### eVLP leads to protein tyrosine phosphorylation through LSECtin/DAP12 complex

**Fig 4:**
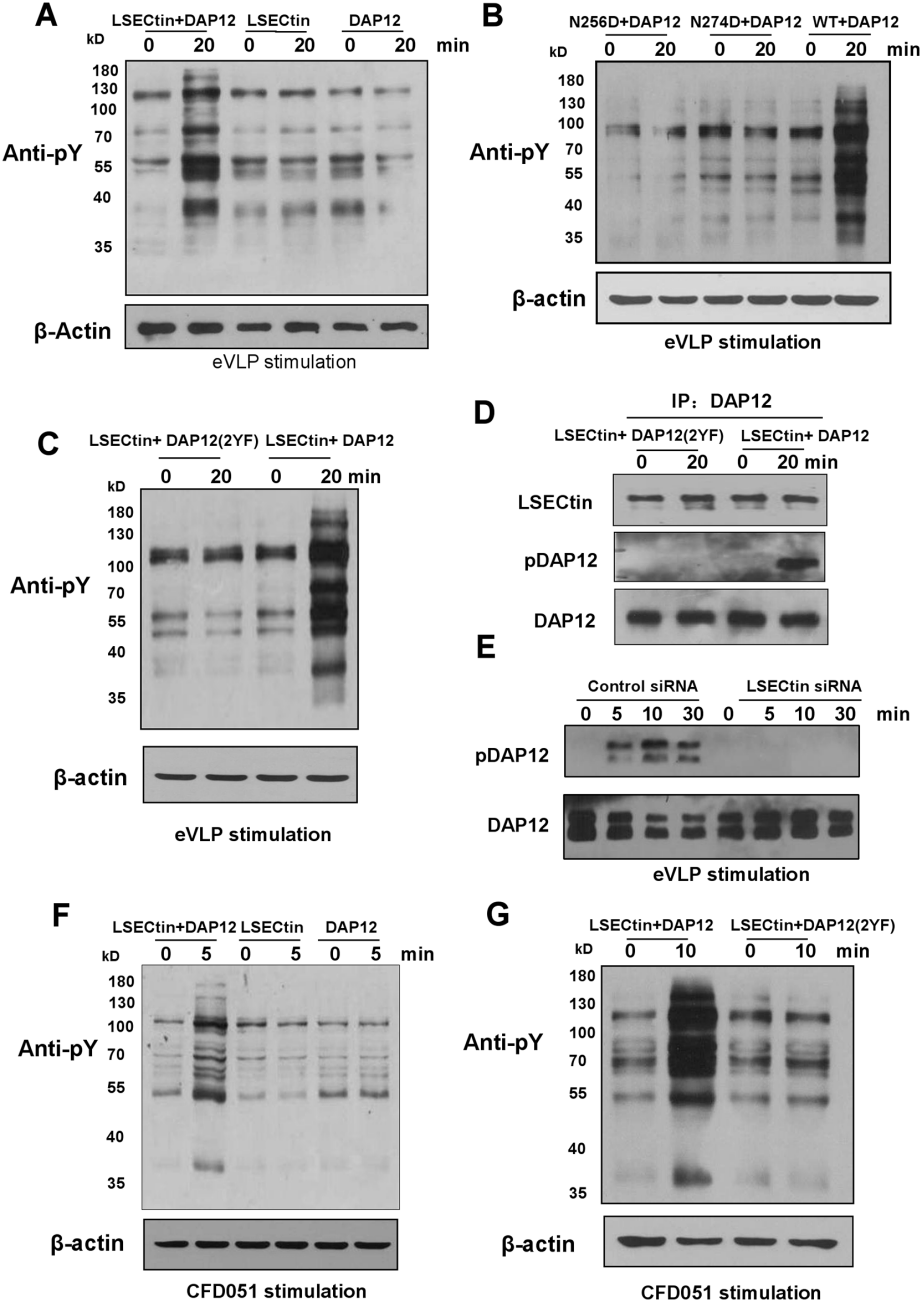
eVLP transduces signals through LSECtin/DAP12 complex. (A) Jurkat cells transfected with LSECtin alone, DAP12 alone or both LSECtin and DAP12 were stimulated with eVLPs for 20min. The cells were lysed and analyzed using anti-phosphotyrosine (4G10). (B) Jurkat-DAP12 stable cells transfected with WT LSECtin or mutant LSECtin (N256D or N274D) were stimulated with eVLPs for 20 min. The cells were lysed and analyzed using anti-phosphotyrosine (4G10). (C, D) Jurkat-LSECtin stable cells transfected with WT DAP12 or ITAM-mutant DAP12 (2YF) were stimulated with eVLPs for indicated times. The cell lysates were analyzed using anti-phosphotyrosine (4G10) (C) or immunoprecipitated with anti-DAP12 antibody. DAP12 phosphorylation was determined by western blotting with anti-phosphotyrosine (4G10) (D). (E) eVLP-induced DAP12 phosphorylation in MDDCs transfected with control siRNA or with LSECtin-specific siRNA was determined by Western blotting as described in (D). (F) Jurkat cells transfected with LSECtin alone, DAP12 alone or both LSECtin and DAP12 were stimulated with plate-bound CFD051 mAb for indicated times. The cells were lysed and analyzed using anti-phosphotyrosine (4G10). (G) Jurkat-LSECtin stable cells transfected with WT DAP12 or ITAM-mutant DAP12 (2YF) were stimulated with plate-bound CFD051 mAb for indicated times. The cells were lysed and analyzed using anti-phosphotyrosine (4G10).

LSECtin binds Ebola GP and is required for eVLP-induced cytokine production. To determine whether eVLPs were able to induce tyrosine kinase-based intracellular signals through LSECtin, Jurkat cells were transfected with LSECtin and DAP12. Jurkat cells do not express LSECtin and DAP12 and refractory to Ebola GP-mediated infection [28]. The LSECtin- and DAP12-transfected cells were either left unstimulated or stimulated with eVLP. Whole-cell extracts were subjected to Western blotting using an anti-phosphotyrosine Ab (4G10) to detect tyrosine-phosphorylated proteins. Compared with correspondingly stimulated LSECtin or DAP12 transfectants, eVLP-treated LSECtin-DAP12 cells yielded increased amounts of tyrosine-phosphorylated proteins (Fig 4A). LSECtin bearing the two amino acid mutants (N256D andN274D) does not bind Ebola GP1-Fc. Consistent with this observation, LSECtin mutants (N256D and N274D) did not deliver an activation signal in response to eVLP stimulation, suggesting that LSECtin recognizes its ligand dependently of Ca^2+^-binding sites (Fig 4B).

Signaling through DAP12 is mediated by its ITAM, which relies on phosphorylation of the two tyrosines within the ITAM for propagation of a signal [29]. To determine whether the two tyrosines of DAP12 are required for LSECtin/DAP12-mediated phosphorylation of protein tyrosines, we transduced a lentivirus encoding wild-type (WT) or mutant DAP12 in which the ITAM tyrosines at positions 91 and 102 were mutated to phenylalanine (2YF) into Jurkat-LSECtin stable cells. Our results show that eVLP stimulation enhanced the phosphorylation of protein tyrosines in Jurkat-LSECtin cells expressing WT but not mutant DAP12 (Fig 4C), suggesting that the two tyrosines within the ITAM are required for LSECtin/DAP12-mediated phosphorylation of protein tyrosines. We next determine whether ligation of LSECtin results in tyrosine phosphorylation of DAP12. Jurkat-LSECtin/DAP12 and Jurkat-LSECtin/DAP12(Y2F) stable cells were stimulated with eVLP. To detect DAP12 phosphorylation, DAP12 protein was immunoprecipitated and tyrosine phosphorylation of DAP12 was examined with 4G10 antibody. Our results show that eVLP induced DAP12 phosphorylation in Jurkat-LSECtin cells expressing WT but not mutant DAP12, although mutant DAP12 can also co-precipitate LSECtin (Fig 4D). More importantly, MDDCs stimulated with eVLP were found to induce DAP12 phosphorylation, but knockdown of LSECtin resulted in a substantial decrease in DAP12 phosphorylation (Fig 4E). In addition, the phosphorylation of DAP12 is independent on TLR4 activation as the effect is not affected after TLR4 “knockdown” in MDDCs (S11 Fig). These results suggest that eVLP-triggered DAP12 phosphorylation is mediated through LSECtin.

In addition, we also used plate-coated anti-LSECtin CFD051 mAb to stimulate LSECtin- and DAP12-transfected cells. This treatment also increased the phosphorylation of protein tyrosines in Jurkat cells expressing LSECtin and DAP12 (Fig 4F). And the enhanced phosphorylation of protein tyrosines is dependent on the two tyrosines within the ITAM of DAP12 (Fig 4G). These results indicate that ligation of LSECtin can induce tyrosine kinase-based intracellular signals in the presence of DAP12.

### Syk tyrosine kinase is essential for eVLP, eVLPm or GP1-Fc-mediated proinflammatory cytokine production

**Fig 5:**
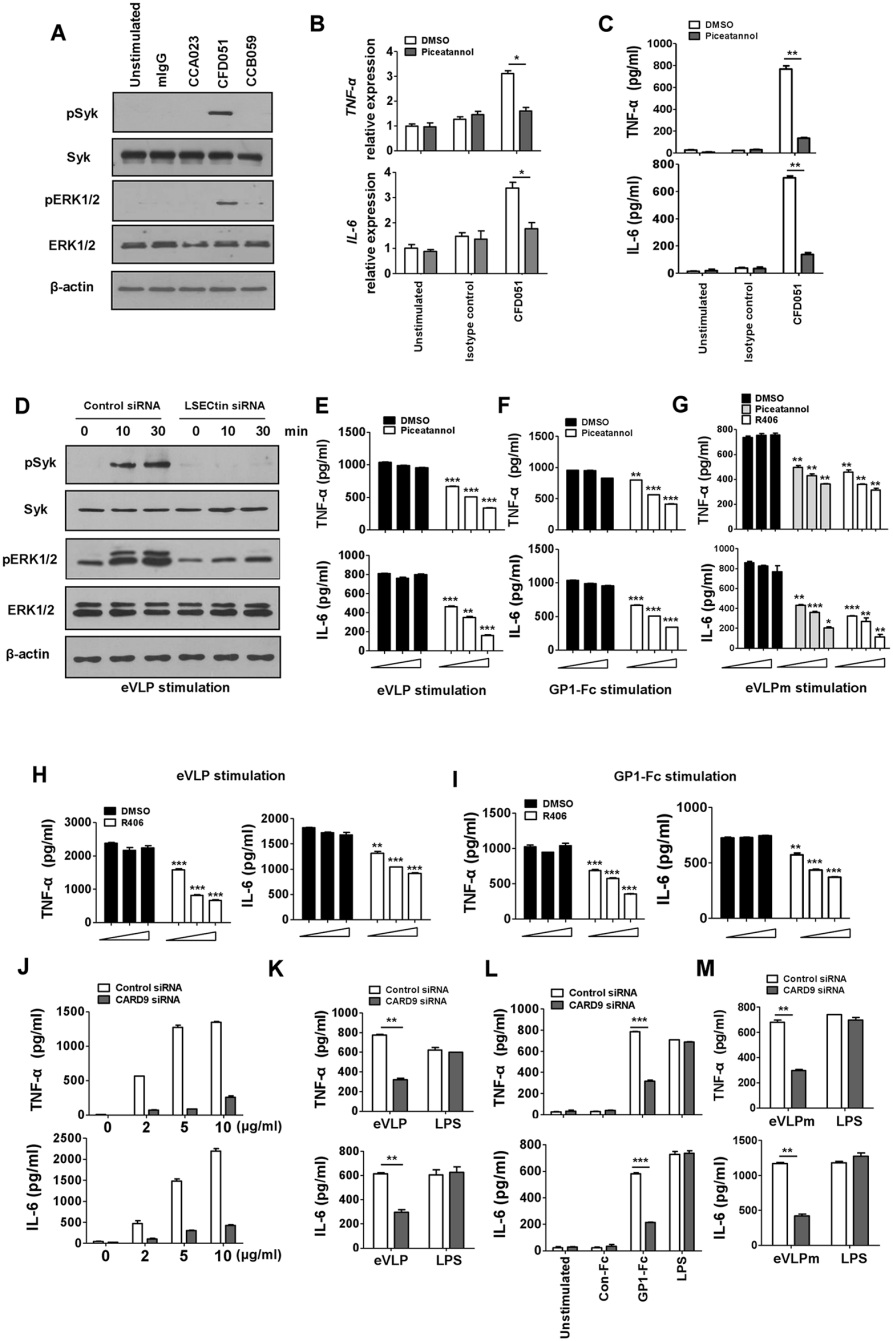
eVLP, eVLPm or GP1-Fc-mediated cytokine production requires Syk kinase activity. (A) Immunoblot of total lysates of MDDCs stimulated with plate-bound mIgG1 isotype control or Abs to LSECtin, including CCA023, CFD051 and CCB059. This immunoblot was probed with Abs to phosphorylated and total Syk and ERK. (B) Real-time RT-PCR analysis of IL-6 and TNF-α from MDDCs stimulated with CFD051 in the presence or absence of piceatannol (10μM). Results are presented as mean ± SD of triplicate wells normalized relative to GAPDH mRNA. (C) MDDCs were stimulated with CFD051 in the presence or absence of piceatannol (10μM). Cytokine production in the supernatants was measured by ELISA after overnight stimulation. (D) MDDCs were transfected with control siRNA or with LSECtin-specific siRNA for 48h and then stimulated with eVLPs for the indicated times. The cell lysates were immunoblotted with Abs to phosphorylated and total Syk and ERK. (E, F) MDDCs were stimulated with eVLPs (E) or GP1-Fc (F) after pre-incubation with either piceatannol (5, 10, or 20μM) or DMSO. (G) MDDCs were stimulated with eVLPm in the presence or absence of piceatannol (5, 10, or 20μM) or R406 (1, 2, or 5μM). Cytokine production in the supernatants was measured by ELISA after overnight stimulation. eVLP, Ebola VLPs produced in insect cells; eVLPm, Ebola VLPs produced in mammalian 293T cells. (H, I) MDDCs were stimulated with eVLPs (H) or GP1-Fc (I) after pre-incubation with either R406 (1, 2, or 5μM) or DMSO. Cytokine production in the supernatants was measured by ELISA after overnight stimulation. (J) MDDCs were transfected with control siRNA or with CARD9-specific siRNA for 48h and then stimulated with control mIgG1 control or CFD051. After stimulation, the cytokine production in the supernatants was measured by ELISA. (K, L and M) MDDCs were transfected with control siRNA or with CARD9-specific siRNA for 48h and then stimulated with eVLP (K), GP1-Fc (L) or eVLPm (M). After stimulation, the cytokine production in the supernatants was measured by ELISA. Data are represented as mean ± SD. Each experiment was repeated with three times with similar results. *p < 0.05; **p < 0.01; ***p < 0.001.

The ITAM in intracellular domain of DAP12 can be phosphorylated and transduce signaling via inducing the phosphorylation of Syk. To determine whether endogenous LSECtin could activate the Syk tyrosine kinase, we stimulated MDDCs with plate-bound anti-LSECtin mAb. Consistent with the results shown in Fig 2A, only plate-bound CFD051 Ab induced phosphorylation of the kinases Syk and ERK in MDDCs (Fig 5A). In addition, the Syk inhibitor piceatannol abrogated the expression of cytokines in MDDCs induced by CFD051 in mRNA and protein levels (Fig 5B and 5C). Identical results were achieved with interfering RNAs (siRNAs) against Syk (S12 Fig), confirming specificity of the Syk inhibitor. Syk inhibition by piceatannol also abrogates the enhanced expression of TNF-α and IL-6 by LSECtin-TLR4 cross-talk (S13 Fig). Considering that another C-type lectin, DC-SIGN, has been shown to mediate signal transduction through Raf-1 [21], we investigated whether Raf-1 is also involved in LSECtin-mediated signaling. The Raf inhibitor GW5074 did not inhibit the expression of cytokines in MDDCs induced by CFD051 (S14 Fig). We next determined whether ligation of LSECtin by eVLP leads to the activation of Syk and ERK. We found that the activation of Syk and ERK induced by eVLP was significantly impaired in LSECtin “knockdown” DCs (Fig 5D). In addition, the phosphorylation of ERK induced by eVLP was also significantly impaired in TLR4 “knockdown” DCs, but the activation of Syk is independent on TLR4 signaling (S15 Fig). These results suggest that eVLP activates Syk through a LSECtin-dependent way. We then examined whether Syk is involved in cytokine expression induced by eVLP. Syk inhibition by piceatannol reduced the production of TNF-α and IL-6 by DCs stimulated with eVLP (Fig 5E), plate-bound GP1-Fc (Fig 5F) or eVLPm (Fig 5G). R406 is another specific Syk inhibitor and in clinical trials for human inflammatory diseases [30]. In the presence of R406, the production of TNF-α and IL-6 by DCs is also significantly suppressed after the stimulation by eVLPs (Fig 5H), plate-bound GP1-Fc (Fig 5I) or eVLPm (Fig 5G). As a control, LPS does not activate Syk kinase and the cytokine production induced by LPS is not suppressed in the presence of either piceatannol or R406 (S16 Fig), which suggests that the inhibition of Syk signaling by piceatannol or R406 is specific to the eVLP and GP1-Fc treatment.

The adaptor molecule CARD9 is required for Syk-mediated inflammatory responses [31]. We therefore investigated the role of CARD9 in LSECtin-mediated responses in MDDCs transfected with siRNA specific for CARD9 or with control siRNA (S17 Fig). We found that LSECtin failed to induce the production of TNF-α and IL-6 in CARD9 “knockdown” MDDCs after treatment with CFD051 antibody (Fig 5J). More importantly, the production of TNF-α and IL-6 by DCs is significantly reduced in CARD9 “knockdown” MDDCs after the stimulation by eVLPs (Fig 5K), plate-bound GP1-Fc (Fig 5L) or eVLPm (Fig 5M). Taken together, these results indicate LSECtin engagement is capable of activating Syk and downstream signaling pathways in DCs, leading to the production of cytokines.

## Discussion

Here, we show that the myeloid C-type lectin receptor LSECtin is a DAP12-coupled activating receptor that induces inflammatory responses by recognizing EBOV GP. LSECtin crosslinked by mAb or ligated with EBOV GP induces the phosphorylation of protein tyrosines and up-regulates the expression of proinflammatory cytokines via Syk and CARD9. Transduction of these events is dependent on the interaction between LSECtin and DAP12, which bears an ITAM in its cytoplasmic domain.

Signaling transduced by some members of the CLRs is crucial for tailoring immune responses to pathogens [32]. To investigate the role of CLRs in regulation of myeloid cell function, mAbs to selectively trigger surface receptors has been widely used, which provides important insight into the signaling and function of different CLRs [33]. We used mAbs to selectively crosslink LSECtin, inducing Syk- and CARD9-dependent inflammatory cytokine production in DCs. It is noteworthy that the anti-LSECtin mAbs failed to induce cytokine production by DCs after transfection with LSECtin siRNA, which further confirms the specificity of the anti-LSECtin mAbs. Thus, LSECtin signaling by itself is sufficient to induce activation of the Syk/CARD9 pathway and gene expression. We also observed that anti-LSECtin mAb treatment combined with LPS enhanced the production of TNF-α and IL-6 by DCs, which indicates that LSECtin might regulate TLR signaling.

C-type lectins comprise a heterogeneous group of transmembrane proteins that recognize various self- and non-self-ligands [19]. These characteristics of CLRs increase the host’s flexibility in recognizing various molecular patterns, including those in exogenous pathogens and endogenous ligands. Our previous studies showed that LSECtin binds activated T cells and inhibits their function through an unidentified endogenous ligand [34]. However, the function of LSECtin as a PRR is still undefined. We have shown here that LSECtin recognizes Ebola GP and transduces an activating signal in DCs. This is contrary to DC-SIGN-mediated immunomodulatory function. For example, DC-SIGN was employed by measles virus to suppress antiviral type I IFN responses and then escape antiviral immunity [35]. It is noteworthy that we used eVLPs or plate-coated GP1-Fc to induce the production of proinflammatory cytokines by MDDCs. Previous studies showed that soluble GP1 alone does not induce cytokine production in human macrophages [15], and we confirmed this with MDDCs. The data is different from that soluble shed GP which can induce the secretion of cytokines. Shed GP is a trimer, but GP1-Fc is a monomer in our study (S1 Fig). Therefore, the different structures of soluble shed GP and GP1 maybe cause their varied ability of activating DCs. In addition, sera Lectins especially MBL in FBS used in our stimulation systems might also interfere GP binding DCs since MBL present in human sera is capable of affecting the binding of shed GP to cells [13].

DAP12 contains a cytoplasmic ITAM that recruits Syk and promotes activation of ERK [36, 37]. Piceatannol and R406, two Syk inhibitors, both significantly inhibit the cytokine production induced by eVLPs or plate-coated GP1-Fc. eVLPs trigger protein tyrosine phosphorylation in LSECtin- and DAP12-co-expressing Jurkat cells, and this effect is dependent on the ITAM of DAP12. Alignment of the LSECtin amino acid sequence indicates that 2 amino acids within CRD, Asn256 and Asn274, interact with Ca^2+^ through their carbonyl groups. Recognition of Ebola GP by LSECtin appears to be dependent on carbohydrates, as eVLPs do not trigger protein tyrosine phosphorylation in mutant LSECtin^N256D or N274D^- and DAP12-co-expressing Jurkat cells. These results show that LSECtin is a novel DAP12-coupled myeloid CLR that acts as a PRR for Ebola GP.

Fatal EBOV infection in humans is associated with severe immune dysregulation and the hypersecretion of numerous proinflammatory cytokines. Recently, it has been demonstrated that trimeric shed GP released from virus-infected cells could activate non-infected DCs and macrophages, causing massive release of pro- and anti-inflammatory cytokines [13]. In addition, Qiu *et al*. reported that ZMapp, a blend of three EBOV GP-specific mAbs, protected EBOV-infected nonhuman primates [38]. This protection occurred even when ZMapp administered 5 days after infection, a time at which the clinical signs of disease are apparent. However, the mechanisms by which protection is achieved are unclear [39]. Given that GP participates in the production of numerous proinflammatory cytokines, it is reasonable to speculate that ZMapp not only neutralizes EBOV infection but also inhibits the excessive cytokine storm by blocking the interaction between GP and its PRRs, such as TLR4 and LSECtin. Therefore, therapeutic strategies to inhibit the cytokine storm should be considered during treatment for Ebola infection, especially for the patients with obvious clinical symptoms. In this regard, treatment with anti-GP, anti-TLR4 and anti-LSECtin Abs could be used to reduce the inflammatory responses caused by shed GP and may be helpful to alleviate the septic shock-like syndrome observed with EBOV infection.

## Materials and Methods

### Reagents

mAbs to human LSECtin were established by immunization of Balb/C mice with recombinant LSECtin extracellular domain protein. Three independent clones, CCA023 (IgG2a), CFD051 (IgG1) and CCB059 (IgG2b), were established [34]. The anti-human LSECtin mAb CCB059 (IgG2b) was selected for staining by flow cytometry. The mouse IgG1 isotype control was from R&D Systems (Minneapolis, MN, USA). mAbs against human HLA-DR, CD83 and CD86 were from eBioscience (San Diego, CA, USA); mAbs against human CD40 and CD80 were from Biolegend (San Diego, CA, USA); anti-phosphotyrosine Ab (4G10) was from Millipore; anti-DAP12 and the other phospho-specific Abs were from Cell Signaling Technology (Danvers, MA). The Syk inhibitors piceatannol and R406 were purchased from Calbiochem (San Diego, CA, USA) and Selleckchem (Houston, TX, USA) respectively. Raf-1 inhibitor GW5074 was purchased from Calbiochem (San Diego, CA, USA); The MyD88 inhibitory peptide Pepinh-MYD was from InvivoGen (San Diego, CA, USA). The GlcNAc β1-2Man disaccharide was purchased from Dextra Laboratories (Reading, UK).

### Purification of recombinant Ebola GP1-Fc protein

The Ebola GP1 coding sequence used is from the GP gene of the Zaire EBOV strain Mayinga (GenBank accession no. AF272001), which contains eight adenosine (A) residues at the editing site. The coding sequence was synthesized by TSINGKE Biological Technology. The GP1 cDNA was cloned by PCR and inserted into pIRES2-EGFP-Fc vectors such that the recombinant protein contained the Fc portion of human IgG. The pIRES2-EGFP-GP1-Fc plasmid was transfected into 293T cells, and the supernatants (free of FBS) were collected for protein purification using protein A/G agarose (GE Healthcare). To determine the content, purified GP1-Fc was subjected to Coomassie blue staining and Western blotting.

### eVLP production in insect Sf9 or mammalian 293T cells

The generation of Ebola VLPs in insect cells (eVLP) has been described previously [26]. Briefly, recombinant baculoviruses co-expressing Ebola VP40 and GP (rBV-GP-VP40) proteins or only expressing Ebola VP40 (rBVVP40) proteins infect *Spodoptera frugiperda* Sf9 insect cells at an MOI of 1. After 48h, the supernants were collected and VP40 and eVLPs proteins were purified in a discontinuous sucrose gradient (10–50%). A visible band between the 30% and 50% sucrose layers was harvested, concentrated by ultracentrifugation and then resuspended in PBS.

Ebola VP40 and GP genes were cloned into pIRES2-EGFP. Mammalian 293T cells were transfected with pIRES2-EGFP-VP40 alone or in combined with pIRES2-EGFP-GP expression vectors at equal DNA concentrations. 48h post-transfection, the supernatants (free of FBS) were collected and clarified with a cell spin. VLPs were purified by centrifugation through a sucrose cushion at 26000 rpm in a Beckman SW-28 rotor for 2 h at 4°C. eVLPs were resuspended in PBS. VP40 and eVLPs containing VP40 and GP proteins produced in mammalian 293T cells was designated VP40m and eVLPm respectively. The final concentration of eVLP protein was quantitated using the DC protein assay (Bio-Rad, Hercules, CA).

### MDDC induction and stimulation

Human peripheral blood mononuclear cells (PBMCs) were isolated from buffy coats from healthy donors using a Ficoll-Paque Plus (GE Healthcare, Piscataway, NJ) gradient. Monocytes were purified from the PBMCs by adherence for 1h at 37°C in complete medium and were differentiated into MDDCs in the presence of 800U/ml GM-CSF and 400U/ml IL-4 (PeproTech). The DCs were stimulated with plate-bound anti-LSECtin mAb, eVLPs eVLPm or plate-bound GP-Fc (10μg/ml) for the indicated times and then lysed and subjected to Western blotting to detect the phosphorylation of Syk and ERK.

### Quantitative real-time PCR

RNA was isolated with RNAeasy Mini Kit (Qiagen, Valencia, CA) and cDNA was synthesized with First Strand cDNA Synthesis Kit (Fermentas). Quantitative PCR was performed with a SYBR Green PCR kit (Roche, Laval, Canada) in an iQ5 (Bio-Rad) detection system. The sequences of the primer pairs of TNF-α, IL-6, CARD9 and TLR4 were described before [40–43]. LSECtin primer pairs were purchased from Qiagen.

### RNA interference

MDDCs were transfected with 20 nM siRNA using the transfection reagent INTERFERin (Polyplus Transfection) as described [44]. Briefly, 5×10^5^ cells were seeded into 6-well plates and then transfected with corresponding siRNAs. After 6 hours, culture medium was replaced with fresh growth medium to reduce cellular toxicity of the transfection reagent. The siRNA sequence was as follows: LSECtin-specific siRNA, 5′-GCGCGAGAACTGTGTCATGAT-3′; DAP12-specific siRNA, 5′- ACAGCGTATCACTGAGACC-3′ [45]; and negative control siRNA, 5′-TTCTCCGAACGTGTCACGTTT-3′. At 48h after transfection, the cells were stimulated. Syk and TLR4 siRNA was purchased from Dharmacon. CARD9 siRNA was purchased from OriGene.

### Construction of expression vectors

The sequence of the gene encoding human LSECtin was obtained from the National Center for Biotechnology Information’s server (GenBank accession no. Q9NY25). LSECtin cDNA was cloned by PCR and introduced into the pcDNA3.1/Myc-His A vector, which has a Myc tag at the N terminus, as did the different LSECtin mutants. Human FceRIγ and DAP12 were inserted into the pCMV-Flag-Mat-1 vector with a Flag tag at the N terminus. To determine how LSECtin associates with DAP12, we constructed different LSECtin mutants. Myc-LSECtin ΔICD lacks the entire intracellular domain (1-31aa). Myc-LSECtin ΔICD&TM lacks the entire intracellular and transmembrane domains (1-55aa). TMΔ1(deletion of 32-43aa), TMΔ2 (deletion of 44-49aa) and TMΔ3 (deletion of 50-55aa) of Myc-LSECtin were confirmed to lack different transmembrane regions, as indicated.

### Statistical analysis

A Student t test was used for statistical analysis. Results with a P value of less than 0.05 were considered as statistically significant.

### Ethics statement

Peripheral blood mononuclear cells (PBMC) are collected from healthy human volunteer donors under approval of Institutional Review Board of Academy of Military Medical Science. The study did not involve any direct contact with human subjects and all samples were anonymized.

## Supporting Information

S1 Fig The identification of purified Ebola GP-Fc protein and binding assay between LSECtin and GP. (A and B) Purified GP1-Fc proteins were analyzed by SDS-PAGE and detected by Coomassie blue staining (A) or immunoblotted with anti-Fc antibodies (B). (C) Purified proteins were analyzed for conformation by noreducing (lane 1) or reducing (lane 2). (D) Binding of LSECtin to plate-bound recombinant GP1-Fc. ELISA plates were coated with 1μg/ml of LSECtin or mutant LSECtin, and then incubated with GP1-Fc at various concentrations. (E) Jurkat stably transfected with WT LSECtin or mutant LSECtin (N256D or N274D) were stained with GP1-Fc (solid line) or Con-Fc (shaded).
(PDF)

S2 Fig LSECtin is selectively expressed on MDDCs. (A) Whole leukocytes were stained with control mIgG2a or anti-LSECtin CCB059 mAb followed by PE-conjugated goat anti-mouse IgG and analyzed by flow cytometry. The high, intermediate and low side-scatter cells correspond to granulocytes, monocytes, and lymphocytes, respectively. (B) Monocytes were induced into MDDCs in the presence of GM-CSF and IL-4. After 6 days of culture, the expression of LSECtin on the MDDCs was identified using the method described in (A). (C and D) Real-time RT-PCR (C) and Immunoblot (D) of LSECtin expression from granulocytes, monocytes, lymphocytes and MDDCs. The results in (C) are presented as the mean ± SD of triplicate wells normalized to GAPDH mRNA.
(PDF)

S3 Fig Immunoblot of LSECtin expression in MDDCs 48h after transfection with LSECtin siRNA. (PDF)

S4 Fig Cytokines production is not induced by the addition of soluble GP1-Fc. MDDCs were stimulated by soluble GP1-Fc (10μg/ml) or LPS as a positive control. Cytokines were detected by ELISA. Data are represented as means±SD of two independent experiments.
(PDF)

S5 Fig MyD88 inhibitory peptide suppresses cytokine production induced by eVLP or plate-bound-GP1-Fc. (A) MDDCs were stimulated with eVLPs after pre-incubation with either Pepinh-MYD (40μM) or control peptide (40μM). Cytokine production in the supernatants was measured by ELISA after overnight stimulation. (B) MDDCs were stimulated with GP1-Fc after pre-incubation with either Pepinh-MYD (40μM) or control peptide (40μM). Cytokine production in the supernatants was measured by ELISA after overnight stimulation. Data are represented as means±SD of two independent experiments. **p < 0.01; ***p < 0.001.
(PDF)

S6 Fig Double silence of TLR4 and LSECtin abrogates the cytokine production iduced by eVLP, eVLPm or plate-bound-GP1-Fc. (A) Real-time RT-PCR analysis of TLR4 expression in MDDCs 24h after transfection with TLR4 siRNA. Results are presented as mean ± SD of triplicate wells normalized relative to GAPDH mRNA. (B) MDDCs transfected with control siRNA, LSECtin siRNA, TLR4 siRNA or LSECtin/TLR4 both siRNA were stimulated with plate-bound GP1-Fc, eVLPs or eVLPm. Cytokine production in the supernatants was measured by ELISA after overnight stimulation. eVLP, Ebola VLPs produced in insect cells; eVLPm, Ebola VLPs produced in mammalian 293T cells. Data are represented as means±SD of two independent experiments. *p < 0.05; **p < 0.01; ***p < 0.001.
(PDF)

S7 Fig The maturation of DCs induced by GP requires LSECtin. MDDCs transfected with control siRNA or LSECtin siRNA were left in medium alone or stimulated with plate-bound GP1-Fc or with LPS (10ng/ml) for 24h. Cell surface expression of CD40, CD80 and CD86 was analyzed by flow cytometry. The data are presented as mean fluorescence intensity (MFI) values.
(PDF)

S8 Fig MyD88 is dispensable for LSECtin signaling. MDDCs were stimulated with immobilized CFD051 mAb or LPS (10ng/ml) after pre-incubation with either Pepinh-MYD (40μM) or control peptide (40μM). Cytokine production in the supernatants was measured by ELISA after overnight stimulation.
(PDF)

S9 Fig LSECtin enhanced the NF-κB activation induced by LPS. Immunoblot of total lysates of MDDCs stimulated with immobilized CFD051 F(ab′)_2_ fragments in the absence or presence of LPS for the indicated times. This immunoblot was probed with Abs to IκBα.
(PDF)

S10 Fig The interaction of LSECtin and DAP12 was independent of the only two hydrophilic threonines (T41 and T42) within the transmembrane region of LSECtin. Immunoblot of HEK293 cells transfected with Flag-tagged DAP12 or mutant (D50A) with substitution of aspartate with alanine together with Myc-tagged LSECtin WT or immunoblot of HEK293 cells transfected with Flag-tagged DAP12 together with Myc-tagged LSECtin mutants with substitution of threonines with alanines (T41A, T42A and T41/42A) after IP with anti-Flag, analyzed by immunoblot with anti-Flag and anti-Myc.
(PDF)

S11 Fig TLR4 is dispensable for DAP12 phosphorylation induced by eVLP. MDDCs transfected with control siRNA or with TLR4-specific siRNA were stimulated with eVLPs. The cell lysates were immunoprecipitated with anti-DAP12 antibody. DAP12 phosphorylation was determined by western blotting with anti-phosphotyrosine (4G10).
(PDF)

S12 Fig Syk silencing abrogates LSECtin-mediated cytokine expression. (A) Immunoblot of Syk expression in MDDCs 48h after transfection with Syk siRNA. (B) MDDCs transfected with control siRNA or with Syk-specific siRNA were stimulated with CFD051. Cytokine production in the supernatants was measured by ELISA after overnight stimulation. Data are represented as means±SD. **p < 0.01; ***p < 0.001.
(PDF)

S13 Fig Piceatannol abrogates the enhanced expression of TNF-α and IL-6 by LSECtin-TLR4 cross-talk. (A) ELISA of TNF-α and IL-6 production by human MDDCs stimulated with LPS in combination with CFD051 in the absence or presence of piceatannol for 18h. Data are represented as means±SD. *p < 0.05.
(PDF)

S14 Fig Raf-1 is not required LSECtin-mediated cytokine expression. Real-time RT-PCR analysis of IL-6 and TNF-α from MDDCs stimulated with CFD051 in the presence of DMSO, piceatannol or Raf inhibitor GW5074. Results are presented as mean ± SD of triplicate wells normalized relative to GAPDH mRNA.
(PDF)

S15 Fig TLR4 is dispensable for Syk phosphorylation induced by eVLP. MDDCs were transfected with control siRNA or with TLR4-specific siRNA for 48h and then stimulated with eVLPs for the indicated times. The cell lysates were immunoblotted with Abs to phosphorylated and total Syk and ERK.
(PDF)

S16 Fig LPS-induced cytokine production is independent on the activation of Syk kinase. (A) MDDCs were stimulated with LPS or eVLP (as a positive control). The cell lysates were immunoblotted with Abs to phosphorylated and total Syk. (B) MDDCs were stimulated with LPS after pre-incubation with either piceatannol (5, 10, or 20μM) or R406 (1, 2, or 5μM). Cytokine production in the supernatants was measured by ELISA after overnight stimulation.
(PDF)

S17 Fig Real-time RT-PCR analysis of CARD9 expression in MDDCs 24h after transfection with CARD9 siRNA. Results are presented as mean ± SD of triplicate wells normalized relative to GAPDH mRNA. Data are represented as means±SD. *p < 0.05.
(PDF)
