# Correction: The Myeloid LSECtin Is a DAP12-Coupled Receptor That Is Crucial for Inflammatory Response Induced by Ebola Virus Glycoprotein

**DOI:** 10.1371/journal.ppat.1005542

**Published:** 2016-03-28

**Authors:** Dianyuan Zhao, Xintao Han, Xuexing Zheng, Hualei Wang, Zaopeng Yang, Di Liu, Ke Han, Jing Liu, Xiaowen Wang, Wenting Yang, Qingyang Dong, Songtao Yang, Xianzhu Xia, Li Tang, Fuchu He

The following information is missing from the Funding section: This study was supported by the National Natural Science Foundation Project (8155100001).
