# Supplementary material for: The Myeloid LSECtin Is a DAP12-Coupled Receptor That Is Crucial for Inflammatory Response Induced by Ebola Virus Glycoprotein
Source: PLoS Pathog. 2016 Mar 4;12(3):e1005487. doi: 10.1371/journal.ppat.1005487 (PMC4778874; doi:10.1371/journal.ppat.1005487)
Supplement: S1 Fig — (A and B) Purified GP1-Fc proteins were analyzed by SDS-PAGE and detected by Coomassie blue staining (A) or immunoblotted with anti-Fc antibodies (B). (C) Purified proteins were analyzed for conformation by noreducing (lane 1) or reducing (lane 2). (D) Binding of LSECtin to plate-bound recombinant GP1-Fc. ELISA plates were coated with 1μg/ml of LSECtin or mutant LSECtin, and then incubated with GP1-Fc at various concentrations. (E) Jurkat stably transfected with WT LSECtin or mutant LSECtin (N256D or N274D) were stained with GP1-Fc (solid line) or Con-Fc (shaded). (PDF) [file ppat.1005487.s001.pdf]

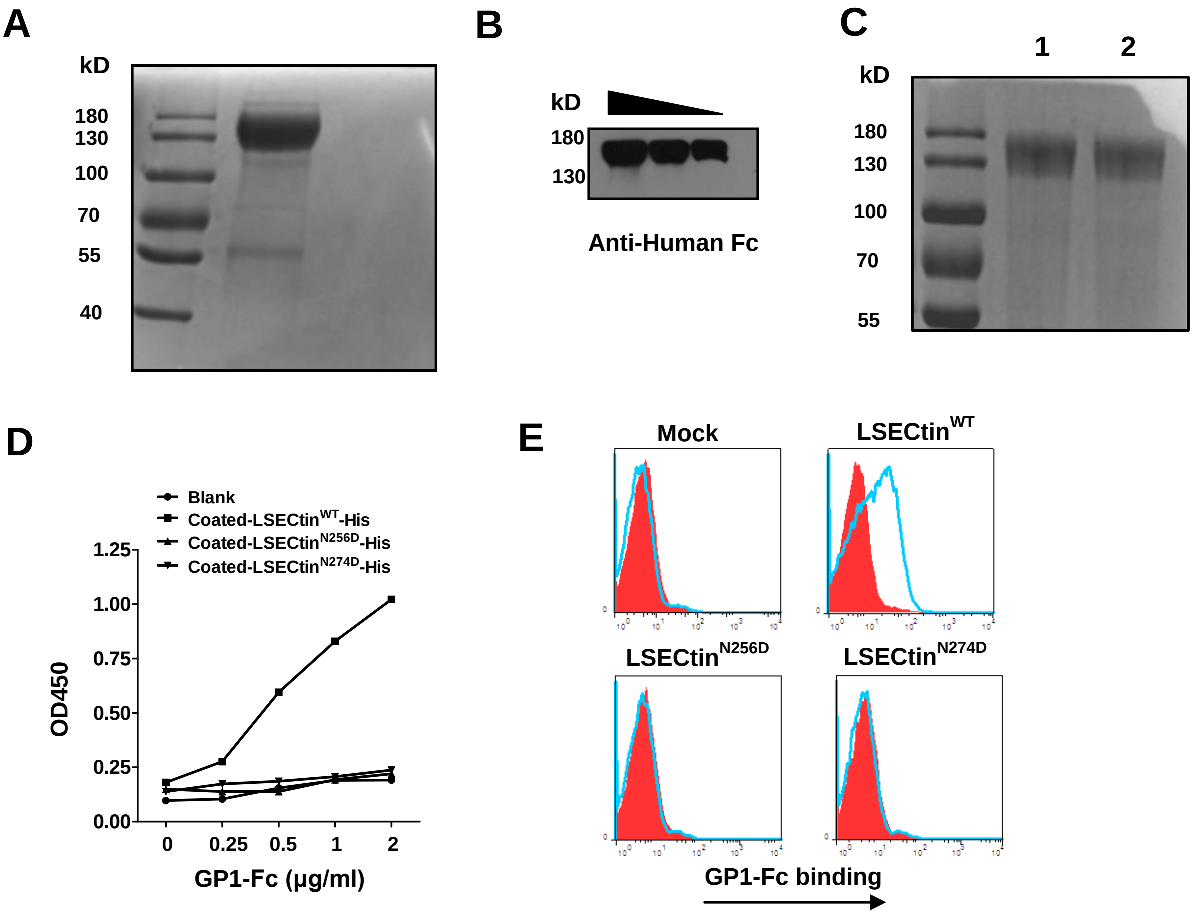

Figure S1. The identification of purified Ebola GP-Fc protein and binding assay between LSECtin and GP.
