# Supplementary figures and images for: The Myeloid LSECtin Is a DAP12-Coupled Receptor That Is Crucial for Inflammatory Response Induced by Ebola Virus Glycoprotein
Source: PLoS Pathog. 2016 Mar 4;12(3):e1005487. doi: 10.1371/journal.ppat.1005487 (PMC4778874; doi:10.1371/journal.ppat.1005487)

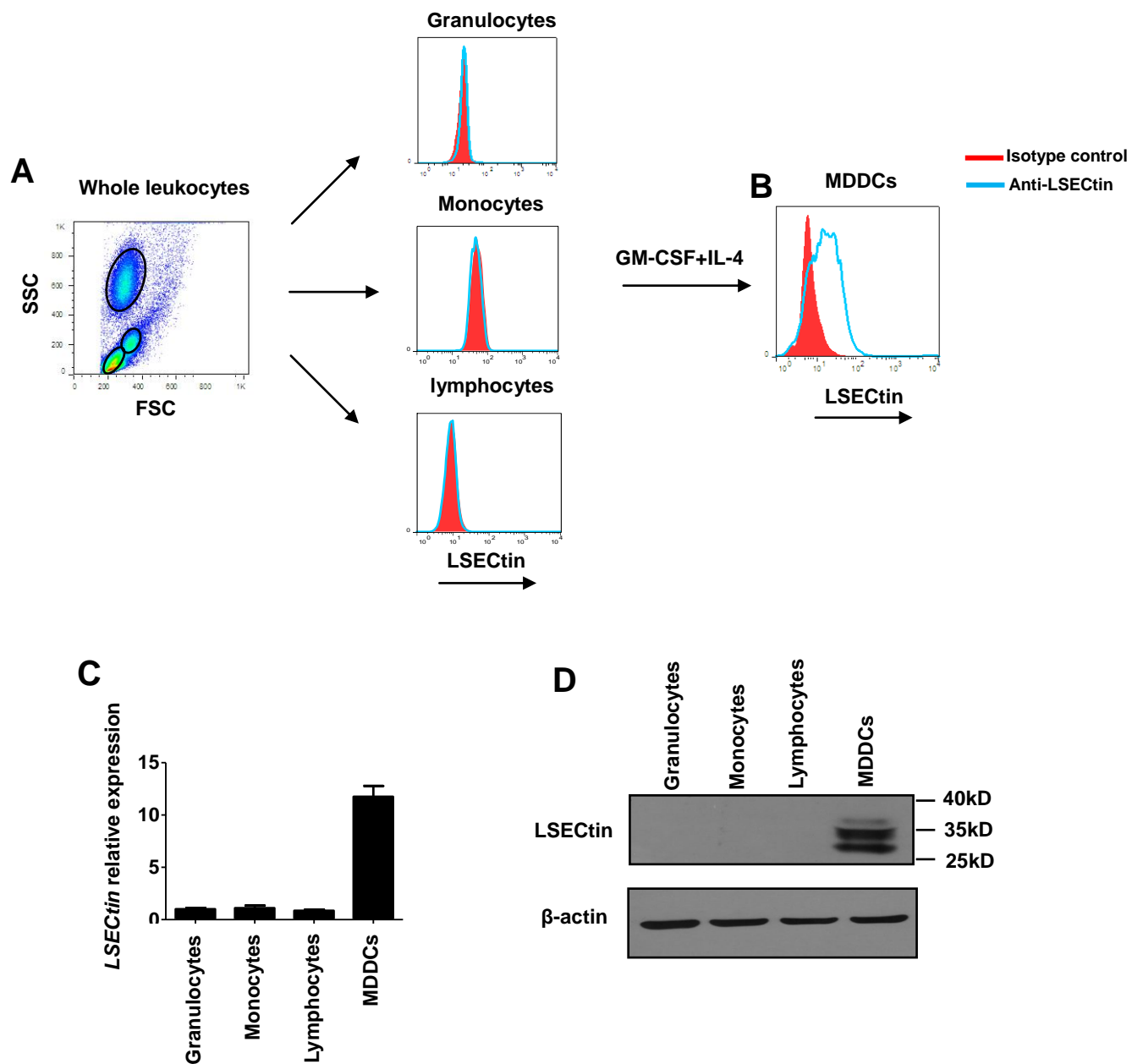

Figure S2. LSECtin is selectively expressed on MDDCs.

Supplement: S2 Fig — (A) Whole leukocytes were stained with control mIgG2a or anti-LSECtin CCB059 mAb followed by PE-conjugated goat anti-mouse IgG and analyzed by flow cytometry. The high, intermediate and low side-scatter cells correspond to granulocytes, monocytes, and lymphocytes, respectively. (B) Monocytes were induced into MDDCs in the presence of GM-CSF and IL-4. After 6 days of culture, the expression of LSECtin on the MDDCs was identified using the method described in (A). (C and D) Real-time RT-PCR (C) and Immunoblot (D) of LSECtin expression from granulocytes, monocytes, lymphocytes and MDDCs. The results in (C) are presented as the mean ± SD of triplicate wells normalized to GAPDH mRNA. (PDF) [file ppat.1005487.s002.pdf]

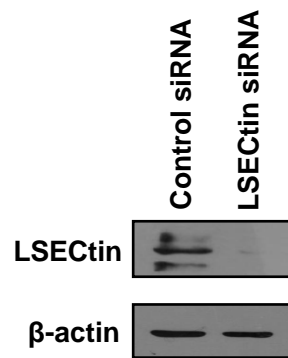

**Figure S3.** Immunoblot of LSECtin expression in MDDCs 48h after transfection with LSECtin siRNA.

Supplement: S3 Fig — (PDF) [file ppat.1005487.s003.pdf]

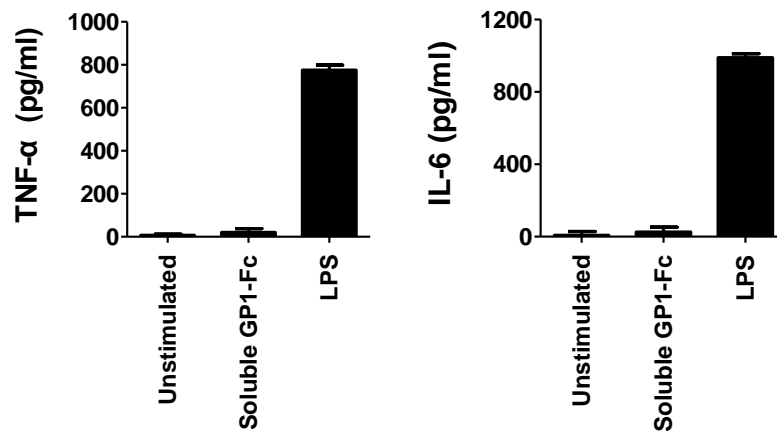

**Figure S4.** Cytokines production is not induced by the addition of soluble GP1-Fc.

Supplement: S4 Fig — MDDCs were stimulated by soluble GP1-Fc (10μg/ml) or LPS as a positive control. Cytokines were detected by ELISA. Data are represented as means±SD of two independent experiments. (PDF) [file ppat.1005487.s004.pdf]

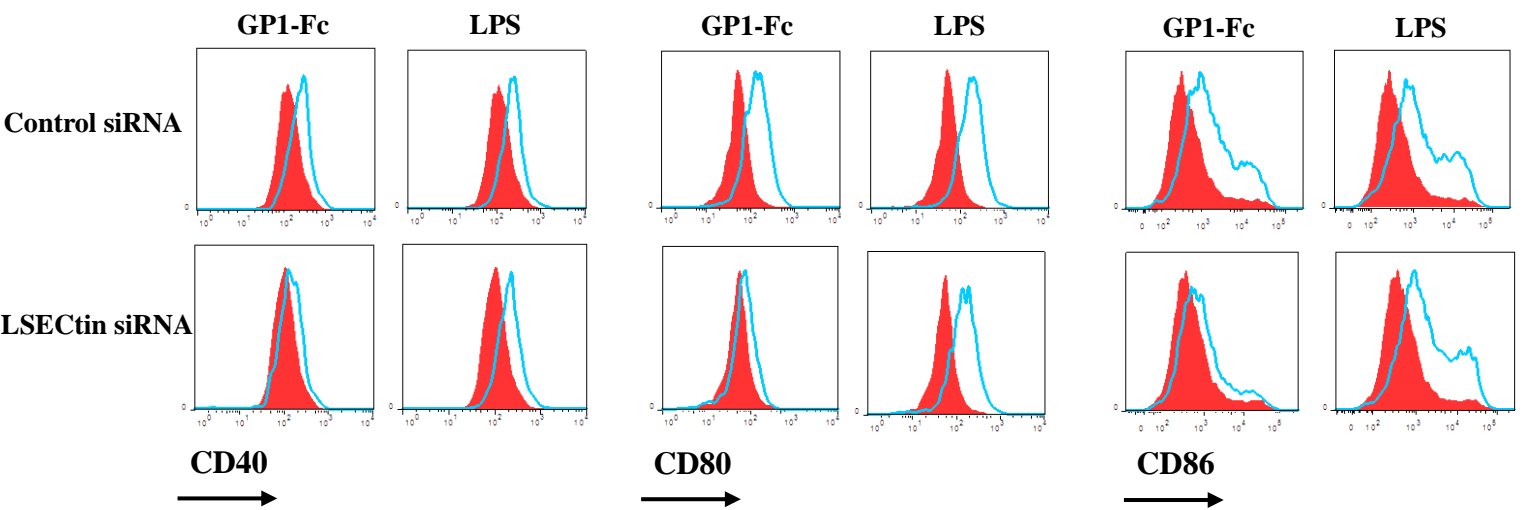

**Figure S7. The maturation of DCs induced by GP requires LSECtin.**

Supplement: S7 Fig — MDDCs transfected with control siRNA or LSECtin siRNA were left in medium alone or stimulated with plate-bound GP1-Fc or with LPS (10ng/ml) for 24h. Cell surface expression of CD40, CD80 and CD86 was analyzed by flow cytometry. The data are presented as mean fluorescence intensity (MFI) values. (PDF) [file ppat.1005487.s007.pdf]

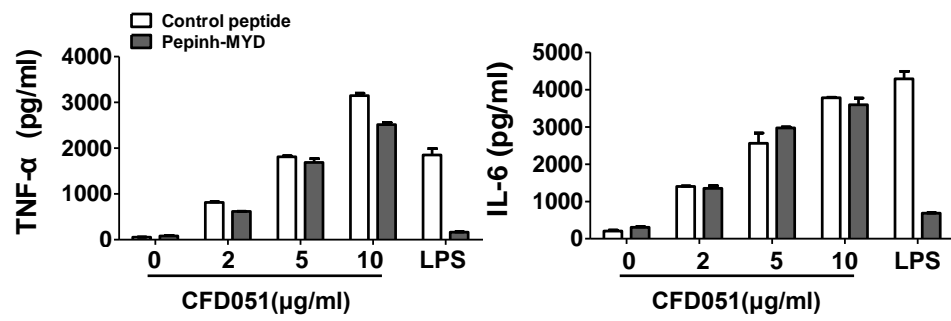

**Figure S8. MyD88 is dispensable for LSEctin signaling.**

Supplement: S8 Fig — MDDCs were stimulated with immobilized CFD051 mAb or LPS (10ng/ml) after pre-incubation with either Pepinh-MYD (40μM) or control peptide (40μM). Cytokine production in the supernatants was measured by ELISA after overnight stimulation. (PDF) [file ppat.1005487.s008.pdf]

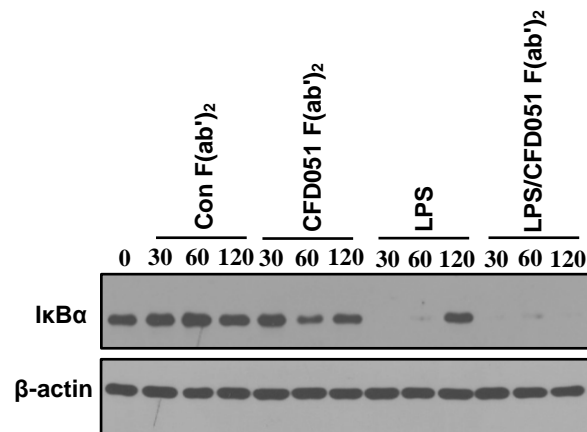

**Figure S9. LSEctin enhanced the NF-κB activation induced by LPS.**

Supplement: S9 Fig — Immunoblot of total lysates of MDDCs stimulated with immobilized CFD051 F(ab′)2 fragments in the absence or presence of LPS for the indicated times. This immunoblot was probed with Abs to IκBα. (PDF) [file ppat.1005487.s009.pdf]

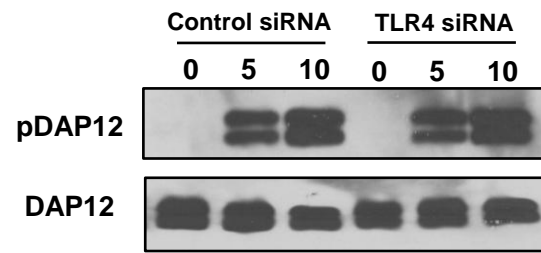

**Figure S11. TLR4 is dispensable for DAP12 phosphorylation induced by eVLP.**

Supplement: S11 Fig — MDDCs transfected with control siRNA or with TLR4-specific siRNA were stimulated with eVLPs. The cell lysates were immunoprecipitated with anti-DAP12 antibody. DAP12 phosphorylation was determined by western blotting with anti-phosphotyrosine (4G10). (PDF) [file ppat.1005487.s011.pdf]

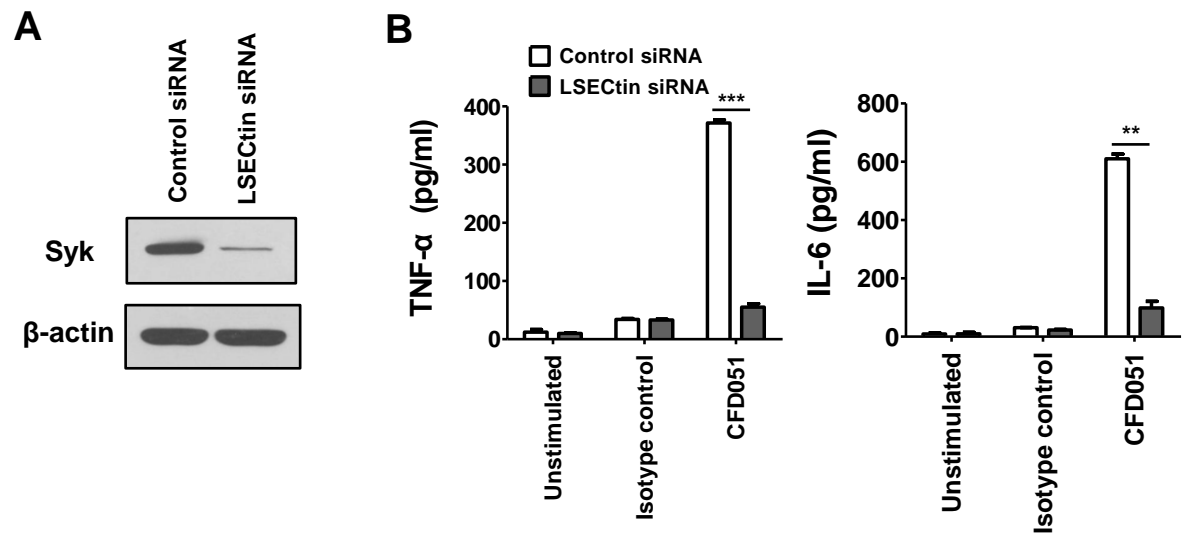

**Figure S12. Syk silencing abrogates LSECtin-mediated cytokine expression.**

Supplement: S12 Fig — (A) Immunoblot of Syk expression in MDDCs 48h after transfection with Syk siRNA. (B) MDDCs transfected with control siRNA or with Syk-specific siRNA were stimulated with CFD051. Cytokine production in the supernatants was measured by ELISA after overnight stimulation. Data are represented as means±SD. **p < 0.01; ***p < 0.001. (PDF) [file ppat.1005487.s012.pdf]

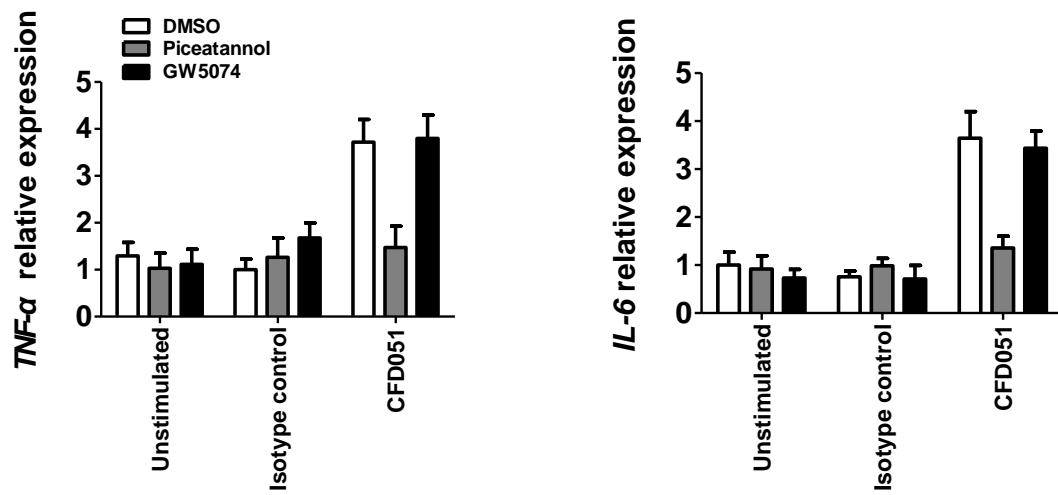

**Figure S14. Raf-1 is not required LSECTin-mediated cytokine expression.**

Supplement: S14 Fig — Real-time RT-PCR analysis of IL-6 and TNF-α from MDDCs stimulated with CFD051 in the presence of DMSO, piceatannol or Raf inhibitor GW5074. Results are presented as mean ± SD of triplicate wells normalized relative to GAPDH mRNA. (PDF) [file ppat.1005487.s014.pdf]

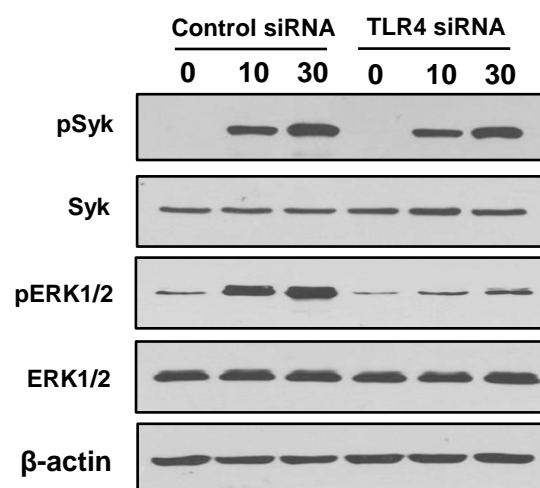

**Figure S15. TLR4 is dispensable for Syk phosphorylation induced by eVLP.**

Supplement: S15 Fig — MDDCs were transfected with control siRNA or with TLR4-specific siRNA for 48h and then stimulated with eVLPs for the indicated times. The cell lysates were immunoblotted with Abs to phosphorylated and total Syk and ERK. (PDF) [file ppat.1005487.s015.pdf]

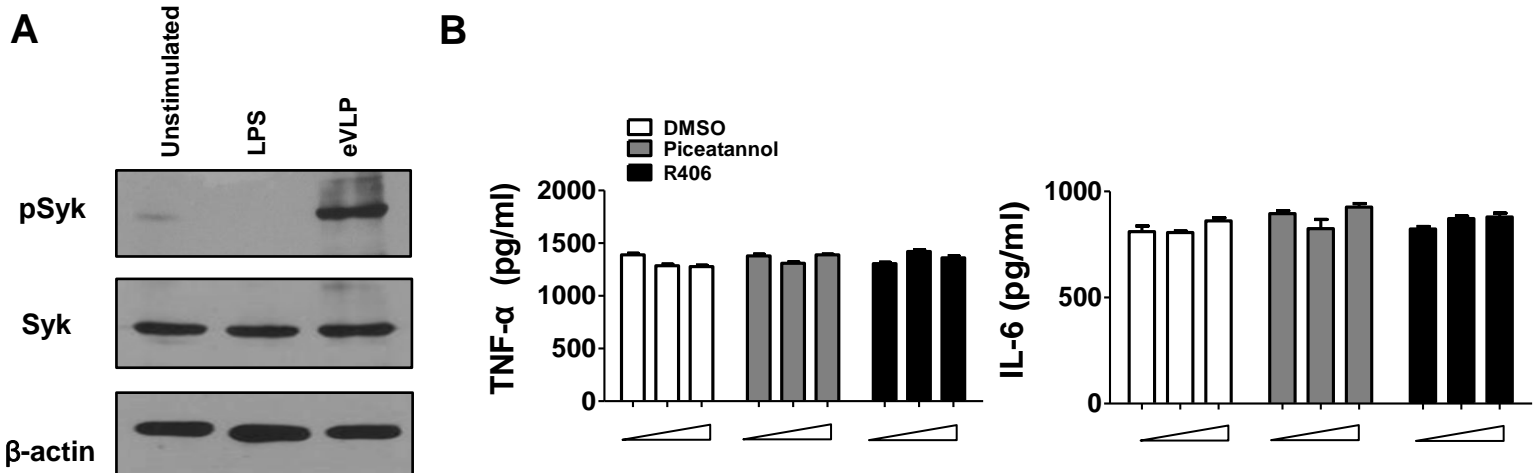

**Figure S16. LPS-induced cytokine production is independent on the activation of Syk kinase.**

Supplement: S16 Fig — (A) MDDCs were stimulated with LPS or eVLP (as a positive control). The cell lysates were immunoblotted with Abs to phosphorylated and total Syk. (B) MDDCs were stimulated with LPS after pre-incubation with either piceatannol (5, 10, or 20μM) or R406 (1, 2, or 5μM). Cytokine production in the supernatants was measured by ELISA after overnight stimulation. (PDF) [file ppat.1005487.s016.pdf]
