# Supplementary material for: The Myeloid LSECtin Is a DAP12-Coupled Receptor That Is Crucial for Inflammatory Response Induced by Ebola Virus Glycoprotein
Source: PLoS Pathog. 2016 Mar 4;12(3):e1005487. doi: 10.1371/journal.ppat.1005487 (PMC4778874; doi:10.1371/journal.ppat.1005487)
Supplement: S5 Fig — (A) MDDCs were stimulated with eVLPs after pre-incubation with either Pepinh-MYD (40μM) or control peptide (40μM). Cytokine production in the supernatants was measured by ELISA after overnight stimulation. (B) MDDCs were stimulated with GP1-Fc after pre-incubation with either Pepinh-MYD (40μM) or control peptide (40μM). Cytokine production in the supernatants was measured by ELISA after overnight stimulation. Data are represented as means±SD of two independent experiments. **p < 0.01; ***p < 0.001. (PDF) [file ppat.1005487.s005.pdf]

A

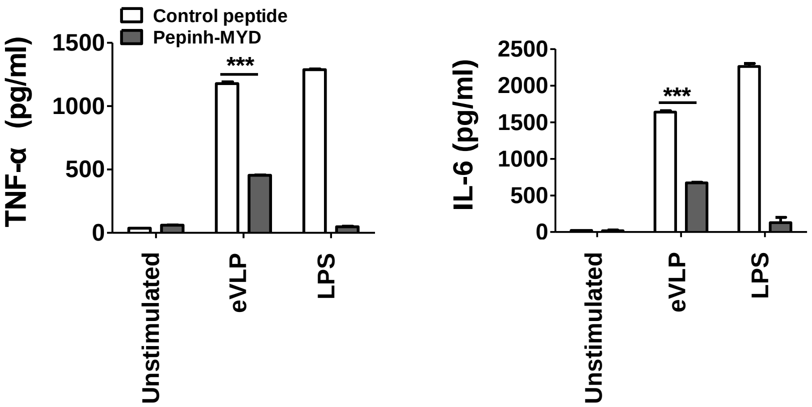

B

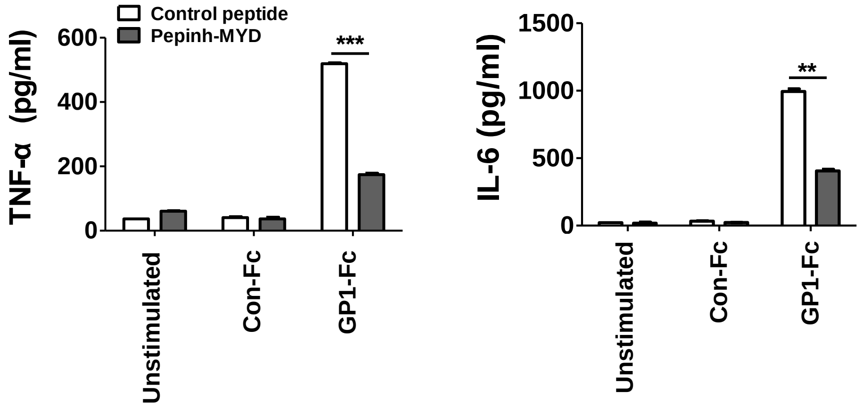

Figure S5. MyD88 inhibitory peptide suppresses cytokine production iduced by eVLP or plate-bound-GP1-Fc.
