# Supplementary material for: The Myeloid LSECtin Is a DAP12-Coupled Receptor That Is Crucial for Inflammatory Response Induced by Ebola Virus Glycoprotein
Source: PLoS Pathog. 2016 Mar 4;12(3):e1005487. doi: 10.1371/journal.ppat.1005487 (PMC4778874; doi:10.1371/journal.ppat.1005487)
Supplement: S6 Fig — (A) Real-time RT-PCR analysis of TLR4 expression in MDDCs 24h after transfection with TLR4 siRNA. Results are presented as mean ± SD of triplicate wells normalized relative to GAPDH mRNA. (B) MDDCs transfected with control siRNA, LSECtin siRNA, TLR4 siRNA or LSECtin/TLR4 both siRNA were stimulated with plate-bound GP1-Fc, eVLPs or eVLPm. Cytokine production in the supernatants was measured by ELISA after overnight stimulation. eVLP, Ebola VLPs produced in insect cells; eVLPm, Ebola VLPs produced in mammalian 293T cells. Data are represented as means±SD of two independent experiments. *p < 0.05; **p < 0.01; ***p < 0.001. (PDF) [file ppat.1005487.s006.pdf]

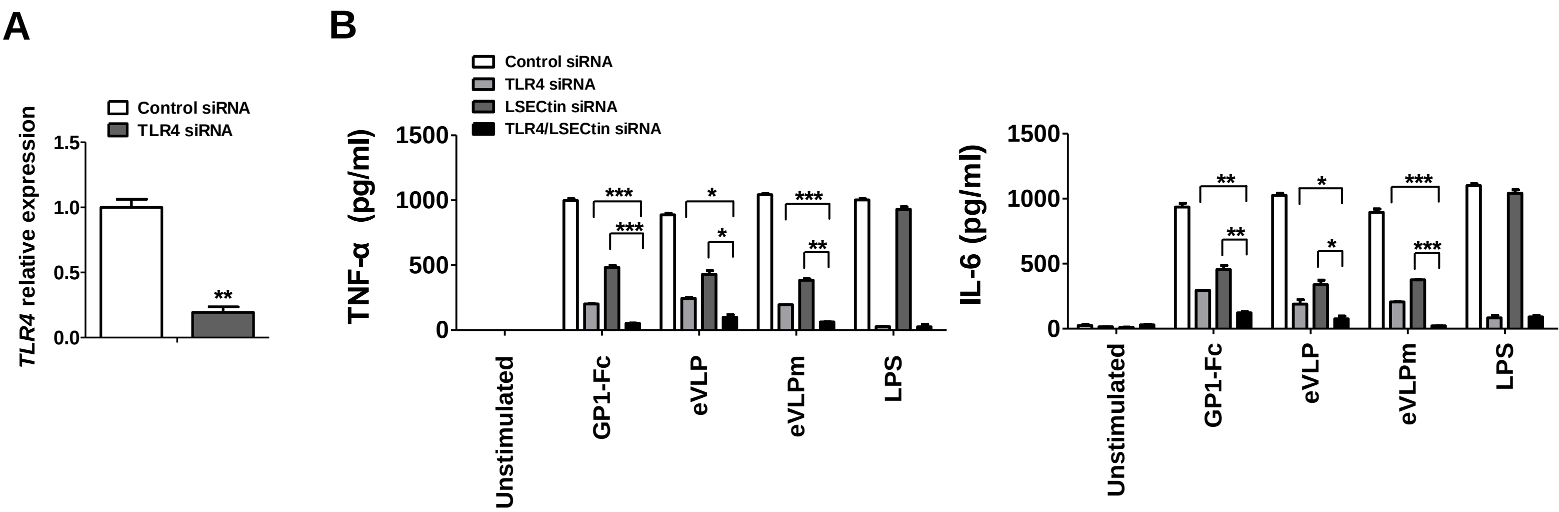

Figure S6. Double silence of TLR4 and LSECtin abrogates the cytokine production iduced by eVLP, eVLPm or plate-bound-GP1-Fc.
