# Supplementary material for: The Myeloid LSECtin Is a DAP12-Coupled Receptor That Is Crucial for Inflammatory Response Induced by Ebola Virus Glycoprotein
Source: PLoS Pathog. 2016 Mar 4;12(3):e1005487. doi: 10.1371/journal.ppat.1005487 (PMC4778874; doi:10.1371/journal.ppat.1005487)
Supplement: S10 Fig — Immunoblot of HEK293 cells transfected with Flag-tagged DAP12 or mutant (D50A) with substitution of aspartate with alanine together with Myc-tagged LSECtin WT or immunoblot of HEK293 cells transfected with Flag-tagged DAP12 together with Myc-tagged LSECtin mutants with substitution of threonines with alanines (T41A, T42A and T41/42A) after IP with anti-Flag, analyzed by immunoblot with anti-Flag and anti-Myc. (PDF) [file ppat.1005487.s010.pdf]

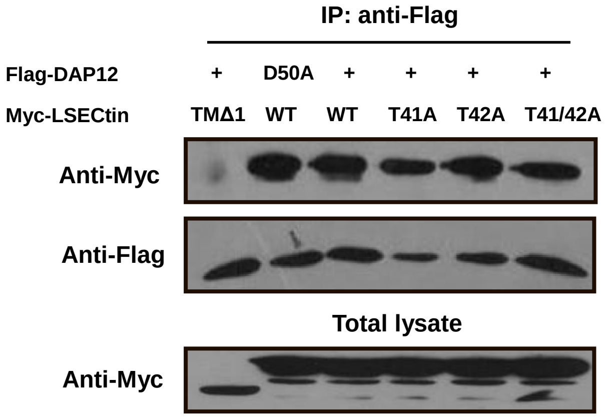

Figure S10. The interaction of LSECtin and DAP12 was independent of the only two hydrophilic threonines (T41 and T42) within the transmembrane region of LSECtin.
