# Supplementary material for: The Myeloid LSECtin Is a DAP12-Coupled Receptor That Is Crucial for Inflammatory Response Induced by Ebola Virus Glycoprotein
Source: PLoS Pathog. 2016 Mar 4;12(3):e1005487. doi: 10.1371/journal.ppat.1005487 (PMC4778874; doi:10.1371/journal.ppat.1005487)
Supplement: S13 Fig — (A) ELISA of TNF-α and IL-6 production by human MDDCs stimulated with LPS in combination with CFD051 in the absence or presence of piceatannol for 18h. Data are represented as means±SD. *p < 0.05. (PDF) [file ppat.1005487.s013.pdf]

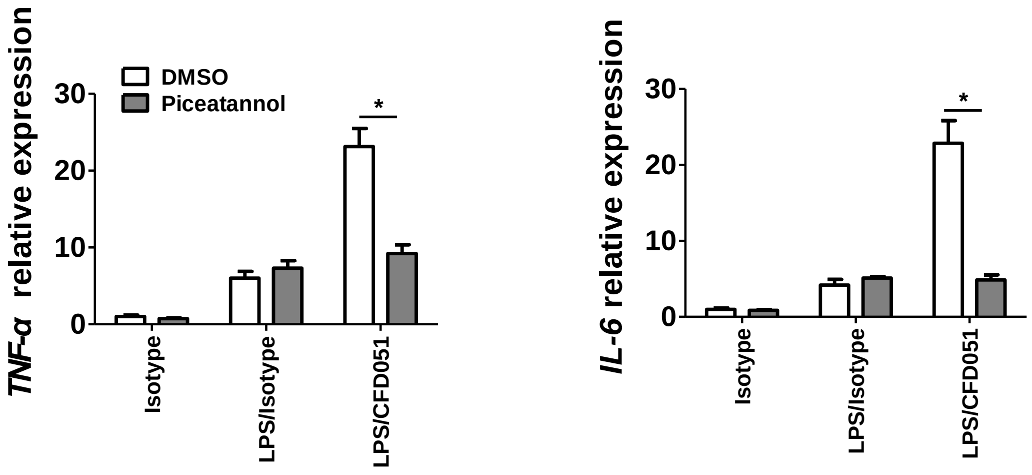

Figure S13. Piceatannol abrogates the enhanced expression of TNF-α and IL-6 by LSECtin–TLR4 cross-talk.
