# Supplementary material for: The Myeloid LSECtin Is a DAP12-Coupled Receptor That Is Crucial for Inflammatory Response Induced by Ebola Virus Glycoprotein
Source: PLoS Pathog. 2016 Mar 4;12(3):e1005487. doi: 10.1371/journal.ppat.1005487 (PMC4778874; doi:10.1371/journal.ppat.1005487)
Supplement: S17 Fig — Results are presented as mean ± SD of triplicate wells normalized relative to GAPDH mRNA. Data are represented as means±SD. *p < 0.05. (PDF) [file ppat.1005487.s017.pdf]

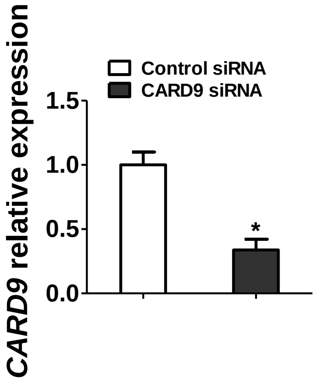

Figure S17. Real-time RT-PCR analysis of CARD9 expression in MDDCs 24h after transfection with CARD9 siRNA.
